# Single-Cell Transcriptomics Reveals Spatial and Temporal Turnover of Keratinocyte Differentiation Regulators

**DOI:** 10.3389/fgene.2019.00775

**Published:** 2019-09-03

**Authors:** Alex Finnegan, Raymond J. Cho, Alan Luu, Paymann Harirchian, Jerry Lee, Jeffrey B. Cheng, Jun S. Song

**Affiliations:** ^1^Department of Physics, Carl R. Woese Institute of Genomic Biology, University of Illinois at Urbana-Champaign, Champaign, IL, United States; ^2^Department of Dermatology, University of California, San Francisco, San Francisco, CA, United States; ^3^Veterans Affairs Medical Center, San Francisco, CA, United States

**Keywords:** Single-cell analysis, transcription regulation, keratinocyte, antioxidant, differentiation

## Abstract

Keratinocyte differentiation requires intricately coordinated spatiotemporal expression changes that specify epidermis structure and function. This article utilizes single-cell RNA-seq data from 22,338 human foreskin keratinocytes to reconstruct the transcriptional regulation of skin development and homeostasis genes, organizing them by differentiation stage and also into transcription factor (TF)–associated modules. We identify groups of TFs characterized by coordinate expression changes during progression from the undifferentiated basal to the differentiated state and show that these TFs also have concordant differential predicted binding enrichment in the super-enhancers previously reported to turn over between the two states. The identified TFs form a core subset of the regulators controlling gene modules essential for basal and differentiated keratinocyte functions, supporting their nomination as master coordinators of keratinocyte differentiation. Experimental depletion of the TFs ZBED2 and ETV4, both predicted to promote the basal state, induces differentiation. Furthermore, our single-cell RNA expression analysis reveals preferential expression of antioxidant genes in the basal state, suggesting keratinocytes actively suppress reactive oxygen species to maintain the undifferentiated state. Overall, our work demonstrates diverse computational methods to advance our understanding of dynamic gene regulation in development.

## Introduction

Keratinocytes, the predominant cell type of mammalian epidermis, regulate their gene expression programs to fulfill specialized cellular functions within the different epidermal strata. Additionally, they must balance self-renewal against cell loss, given the epidermis’ intrinsic replacement rate of ∼28 days in normal human skin. How keratinocytes dynamically govern the hierarchy of self-renewal, differentiation, and maturation remains poorly understood. This article reconstructs the dynamic gene regulatory network rearrangements that occur with keratinocyte differentiation by analyzing human foreskin single-cell RNA-seq (scRNA-seq) data.

Basal keratinocytes (BKs) comprise the basal layer, the innermost layer of the epidermis. Basal keratinocytes divide at controlled rates that are thought to be heterogeneous across progenitor cells, ranging from rarely dividing self-renewing stem cells to rapidly cycling transit amplifying cells (Alcolea and Jones, 2014). In addition to replicating, BKs constitute the basement membrane, which is critical for adhesion of the epidermis and dermis and participate in intercellular signaling required for maintaining tissue homeostasis. Upon differentiation, differentiated keratinocytes (DKs) exit the cell cycle and travel from the basal layer through the more superficial spinous and granular layers culminating in cornification/cell death. During the differentiation process, keratinocytes synthesize components necessary for epidermal barrier function, including desmosomes (specialized adhesion structures) in the spinous layer, secretory organelles called lamellar granules that contain lipids and enzymes, and keratohyalin granules, which contain proteins such as loricrin—the latter two providing vital components of the cornified lipid envelope of the epidermis’ outer stratum corneum layer.

At the transcriptomic level, the stratum-specific expression patterns of many key Keratinocyte Genes are known, but regulators of these genes are still being identified (Lopez-Pajares et al., 2015). Constructing the dynamic regulatory network of relevant transcription factors (TFs) and their target genes thus remains an active area of investigation. Previous studies have used various genomic and epigenomic data to construct regulatory networks. For example, Lopez-Pajares et al. (2015) analyzed the time-series transcriptome of experimentally differentiated keratinocyte cultures and identified regulatory relations of genes based on temporal coexpression patterns. Joost et al. (2016) advanced this approach to the single-cell level in murine epidermis, identifying TFs varying with differentiation pseudotime and constructing gene modules using correlation-based expression similarity. In *in vitro* keratinocyte epigenomic studies, Cavazza et al. (2016) and Klein et al. (2017) mapped typical enhancers and super-enhancers (SEs)—large clusters of enhancers characterized by strong activating histone modifications, enrichment of cell type–specific TF motifs, and regulation of cell type–specific genes (Hnisz et al., 2013). Both works identified dramatic changes in sets of SEs between the BK and DK states and developed regulatory networks based on patterns of TF binding/motif enrichment in SEs and proximities of SEs to gene loci (Cavazza et al., 2016; Klein et al., 2017). More recently, the single-cell Perturb-ATAC method revealed changes in regulatory element chromatin accessibility during keratinocyte differentiation and targeted genetic perturbation (Rubin et al., 2019); these data permitted the grouping of TFs with correlated binding site accessibility during differentiation, the inference of interactions between TFs, and the detection of synergy in perturbations of chromatin accessibility (Rubin et al., 2019).

While regulation by TFs and epigenetic modifications ultimately determine gene expression, changes in redox state and abundance of reactive oxygen species (ROS) may help guide the transition from basal to differentiated states (Bigarella et al., 2014). For instance, Hamanaka et al. (2013) demonstrated that reducing ROS through inhibition of oxidative phosphorylation impairs epidermal differentiation and increases proliferation of basal cells and that treatment of cultured keratinocytes with antioxidants impairs differentiation. Likewise, Bhaduri et al. (2015) established MPZL3 and FDXR as proteins localizing to the mitochondria and inducing keratinocyte differentiation by increasing ROS levels. These findings demonstrate opposing roles of ROS and antioxidants in regulating differentiation; however, a genome-wide time-course examination of genes potentially modulating differentiation *via* their antioxidant function has not yet been described.

In this article, we use our recently generated scRNA-seq data assaying expression in 22,338 human foreskin keratinocytes (Cheng et al., 2018) to identify regulators of keratinocyte differentiation and computationally infer dynamic TF networks controlling gene expression patterns required for keratinocyte development and function. We find that expression turnover of established and predicted keratinocyte regulators coincides with previously reported change in SE sets between the BK and DK states (Klein et al., 2017). Depletion of two predicted positive regulators of BKs—ZBED2 and ETV4—leads to differentiation of BKs in the absence of external differentiation-inducing queues. The pattern of differential TF binding-motif enrichment between BK- and DK-specific SEs follows the pattern of TF state-specific expression, leading us to develop gene regulatory networks for TFs. These networks recapitulate known and previously predicted regulatory relationships and also identify novel regulators of differentiation stage–specific functions. In particular, our predicted regulation of cadherins by ETV4 suggests that ETV4’s established role of controlling cadherin-mediated cell sorting in branches of the neuronal lineage (Livet et al., 2002; Helmbacher, 2018) may extend to keratinocytes. Supporting the role of cellular antioxidants in suppressing ROS levels, we find that genes related to antioxidant function are preferentially expressed in BK cells and also uncover differences in subcellular localization between antioxidant genes exclusively expressed in BK state and those in DK state.

## Results

### A Subset of Keratinocyte-Specific Transcription Factors Shows Expression and Binding Patterns Coupled to State-Specific Epigenomes

To identify expression patterns of key TFs across distinct keratinocyte transcriptomic states, we examined a set of 49 established and 44 candidate Keratinocyte regulators, to which we refer below as Keratinocyte TFs. Established keratinocyte regulators were obtained from a previous publication (Klein et al., 2017); Candidate TFs were identified based on keratinocyte-specific RNA expression in the FANTOM5 (Functional ANnoTation Of the Mammalian genome) cell atlas (Fantom Consortium et al., 2014) (Methods; **Supplementary File 1: Figure S1**; **Supplementary File 2: Tables S1**, **Table S2**). Our approach of selecting candidates based on cell type–specific expression aimed to increase the confidence that changes in TF expression across single-cell transcriptional states reflect rewiring of gene regulatory networks guiding keratinocyte differentiation and to reduce false positives in subsequent identification of TF targets from correlation analysis.

**Figure 1:**
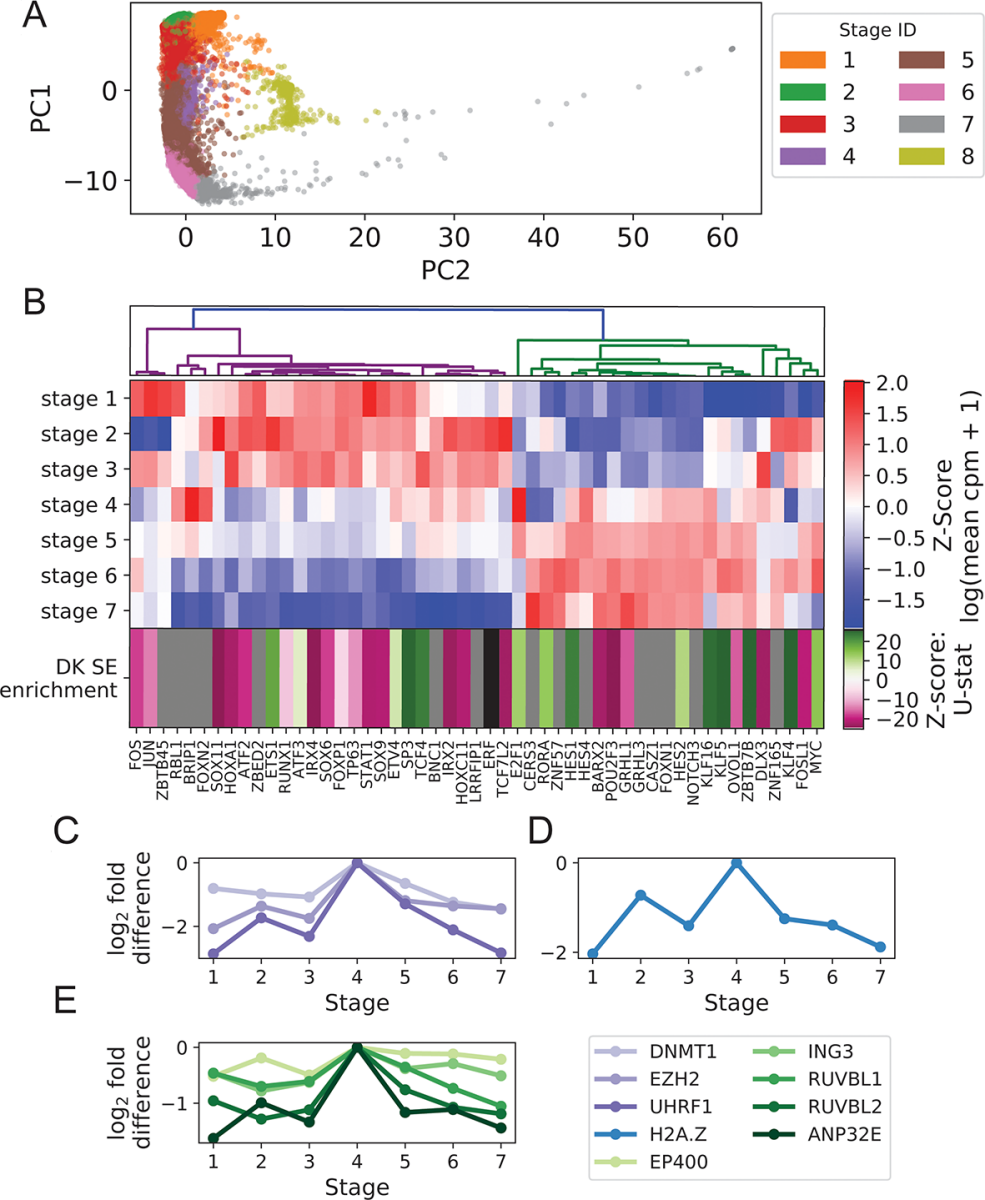
Turnover in Keratinocyte TF expression is temporally and spatially coupled to turnover in SEs. **(A)** Imputed single-cell expression vectors of 22,338 foreskin keratinocytes projected onto first two principal components; stage membership was assigned by k-means–based approximate spectral clustering. **(B)** First seven rows show log-transformed stage-wise mean imputed expression of dynamic Keratinocyte TFs normalized across stages. Bottom row shows the magnitude and direction of differential motif enrichment between BK and DK SEs. Gray and black cells correspond to TFs without a known binding motif and TFs not differentially enriched between SE sets, respectively. Columns are organized by hierarchical clustering on first seven rows (Methods). **(C−E)** Log fold-change in stage-wise mean imputed expression between stage 4 (mitotic state) and other stages for established keratinocyte epigenetic regulators **(C)**, H2A.Z **(D)**, and a subset of components of SWR1 remodeler complex **(E)**. See also **Supplementary File 1: Figures S1−4**.

We clustered foreskin keratinocytes into eight stages *via* approximate spectral clustering of imputed scRNA expression values (**Figure 1A**; **Supplementary File 1: Figure S2**; Methods). As observed previously (Cheng et al., 2018), marker gene expression profiles indicated that these stages largely agreed with known keratinocyte states including a BK state (corresponding to stages 1−3), a mitotic state (stage 4), and a DK state (stages 5−7) (**Supplementary File 1: Figure S3**). The mitotic state had markedly increased levels of cyclins as well as the histone H2A isoform *HIST2H2AC* known to be required for proliferation of undifferentiated mammary epithelial cells (Monteiro et al., 2017). Additionally, the mitotic state had high expression of basal markers (*KRT5, KRT14*) and intermediate expression of early differentiation markers (*KRT1, KRT10*), suggesting it is a rapidly cycling subpopulation in transition from the BK to DK states (**Supplementary File 1: Figure S3**). This interpretation is supported by *in situ* hybridization experiments that have identified basal and suprabasal expression of the mitotic marker gene *MKI67* (Cheng et al., 2018). Stage 8 reproduced the “channel” cluster, identified previously as a novel keratinocyte cell state not on the classic differentiation trajectory (Cheng et al., 2018).

Hierarchical clustering of Keratinocyte TFs that exhibit dynamic expression across stages 1 to 7 clearly separated the TFs with peak expression in the BK state from those with peak expression in the DK state (**Figure 1B**), with a sharp transition occurring in the mitotic state (stage 4). This pattern of expression turnover coincided with the dramatic change in distribution of active SEs between the BK and DK states (previously identified from differential histone modification patterns of H3K4 monomethylation, H3K4 trimethylation and H3K27 acetylation) (Cavazza et al., 2016; Klein et al., 2017). We therefore hypothesized that the TFs with peak expression in each state may function through direct binding of state-specific SEs, thereby coupling the transcriptional and epigenetic developmental programs. To test this hypothesis, we first compared the distributions of TF motif occurrence counts (scaled by SE length) between BK and DK SEs and identified 21 and 14 TFs with motifs significantly differentially enriched between BK and DK SEs, respectively. Next, we assigned to each of these TFs a direction and magnitude of differential motif enrichment (**Figure 1B** last row, **Supplementary File 3**: Clustering transcription factor expression trajectories and super-enhancer differential motif enrichment). Grouping the TFs into two expression clusters as shown in **Figure 1B**, we found that the direction and magnitude of TF differential motif enrichment in BK versus DK SEs generally agreed with each cluster’s peak expression in BK versus DK state (*p* = 0.043, one-sided Mann-Whitney *U* test); intuitively, the left (magenta) and right (green) expression branches in **Figure 1B** contained more magenta and more green boxes, respectively, in the last row of **Figure 1B**. This finding, made possible by single-cell analysis, supported the premise that Keratinocyte TF expression and chromatin conformation accessibility are coordinated during transition between keratinocyte cell states.

Next, we identified potential regulators of the switch in state-specific SEs by examining the stage-wise expression of established keratinocyte epigenetic regulators and found several of them, including *EZH2*, *DNMT1*, and *UHRF1*, to have a strong expression spike in the mitotic state (Ezhkova et al., 2009; Sen et al., 2010) (**Figure 1C**). Additionally, we found that *H2A.Z* and components of the SWR1 remodeling complex, responsible for depositing this enhancer-associated histone subunit, attained peak expression in the mitotic state (**Figures 1D, E**). Although the sharp increase in the expression of *H2A.Z* and other histone subunits in this state may be partially explained by the abundance of rapidly dividing cells, the concurrent peak expression of SWR1 components suggested active reorganization of enhancer activities prior to differentiation. Together, these single-cell results highlighted epigenetic remodelers functioning during the mitotic state, potentially to facilitate the turnover of SEs between the BK and DK states.

### Knockdown of ETV4 and ZBED2, Predicted Promoters of the BK State, Induces Differentiation

To validate the regulatory function of Candidate Keratinocyte TFs, we ranked the TFs based on their predicted ability to promote the BK state. Candidates were assigned a differentiation-promoting score by first identifying highly correlated keratinocyte-specific regulatory targets and summing their log fold changes between DK and BK states, accounting for the sign of correlation (Methods; **Supplementary File 1: Figure S4**). We filtered out TFs with low expression in undifferentiated keratinocyte cultures (<5 FPKM) and knocked down four of the top five remaining TFs with greatest BK-promoting strength (strong negative differentiation-promoting score) using RNAi in the absence of external differentiation queues.

**Figure 2:**
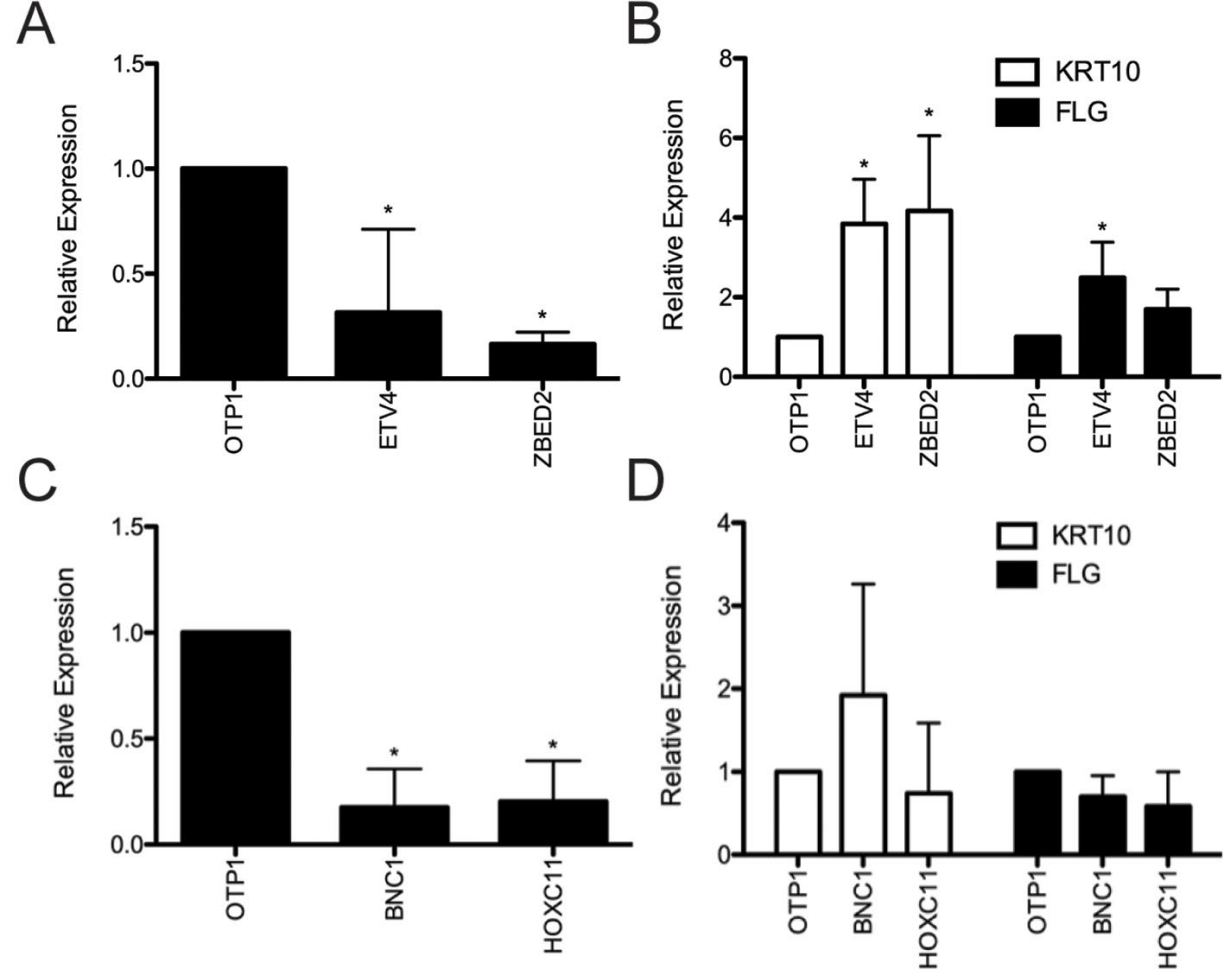
Evaluation of predicted keratinocyte regulators *via* siRNA knockdown. **(A)** RNA was harvested 4 days after transfection from primary human keratinocyte culture treated with *ETV4*, *ZBED2*, or negative control siRNA. Quantitative polymerase chain reaction analysis showed significant (*p* < 0.05, Student *t* test) knockdown of *ETV4* and *ZBED2* mRNA relative to nontargeting siRNA transfected cells. **(B)** Expression of *KRT10* and *FLG* transcript following siRNA knockdown of *ETV4* or *ZBED2*, relative to control. Asterisks indicate *p* < 0.05 (Student *t* test). **(C)** same as **(A)** but for *BNC1* and *HOXC11*. **(D)** Knockdown of *BNC1* and *HOXC11* did not significantly change expression of differentiation marker genes *KRT10* and *FLG*. Error bars indicate 1 standard deviation calculated over four replicates.

Depletion of *ETV4* and *ZBED2* transcripts resulted in a significant increase in mRNA expression of the early differentiation marker *KRT10* by 3.84- and 4.17-fold, respectively, compared with control cells transfected with nontargeting siRNA (**Figures 2A, B**). Depletion of *ETV4* also showed a significant increase (2.49-fold) in the mRNA expression of the late differentiation marker *FLG*, with *ZBED2* depletion also showing a similar trend (**Figure 2B**). These results confirmed the strong progenitor-promoting function of ETV4 and ZBED2, synthetic reduction of which induced spontaneous differentiation of keratinocytes.

Depletion of *BNC1* and *HOXC11* transcripts did not significantly change the mRNA level of *KRT10* or *FLG* (**Figures 2C, D**), suggesting that the regulatory effects of these TFs do not extend to these differentiation markers or that BNC1 and HOXC11 protein expression was not diminished enough to have an effect. Nevertheless, previous knockout of *BNC1* in mouse significantly decreased the number of proliferating keratinocytes in the cornea of the eye (Zhang and Tseng, 2007). Therefore, we conclude that BNC1 likely promotes the BK state in foreskin, although its regulatory targets remain to be experimentally characterized.

Previous reports supported our prediction of the role of SOX9 and IRX4 in keratinocyte differentiation (**Supplementary File 1: Figure S4**). For example, overexpression of SOX9 in keratinocytes has been shown to suppress the late differentiation maker genes IVL and LOR (Shi et al., 2013). Likewise, IRX4 was previously predicted to regulate keratinocyte proliferation and hemidesmosome assembly based on correlation with functionally annotated genes across a large set of publicly available mouse RNA-seq data (Lachmann et al., 2018). Moreover, knockdown of the differentiation-promoting TF GRHL3 in calcium-induced keratinocyte primary cells resulted in a gain of SEs strongly enriched for the IRX4 motif (Klein et al., 2017), suggesting antagonism between IRX4, and this established prodifferentiation TF. Overall, our prioritization of Candidate TFs revealed novel keratinocyte regulators and provided additional candidates for follow-up experiments.

### Gene Modules in the Basal Network Promote Tissue Architecture, Control of Hippo Signaling, and Progression to the Mitotic State

We next sought to assign function to Keratinocyte TFs with motifs enriched in state-specific SEs based on their scRNA-seq expression correlation with a set of potential regulatory targets. This set was composed of the Keratinocyte TFs themselves and an additional 747 genes differentially upregulated in FANTOM5 keratinocytes compared with other cell types (Methods; **Supplementary File 2: Table S2**). Focusing first on the regulatory network governing the BK state and its progression to the mitotic state, we clustered the Keratinocyte TFs with enriched motifs in BK SEs based on their expression similarity across single cells in stages 1 to 4. We then clustered the regulatory targets into gene modules based on the similarity of their correlations to the TFs. Organizing the TF/target correlation matrix by TF and gene modules (**Supplementary File 1: Figure S5A**) yielded submatrices with strong correlation/anticorrelation delineated by module boundaries. Thresholding on the average correlation strength calculated across gene/TF pairs for each TF and gene module, we identified activating and inhibiting relationships between 13 TF and 23 target gene modules (**Supplementary File 1: Figure S5 (B–D)**; **Supplementary File 2: Table S3**; **Supplementary File 3**: Regulatory network construction).

**Figure 3:**
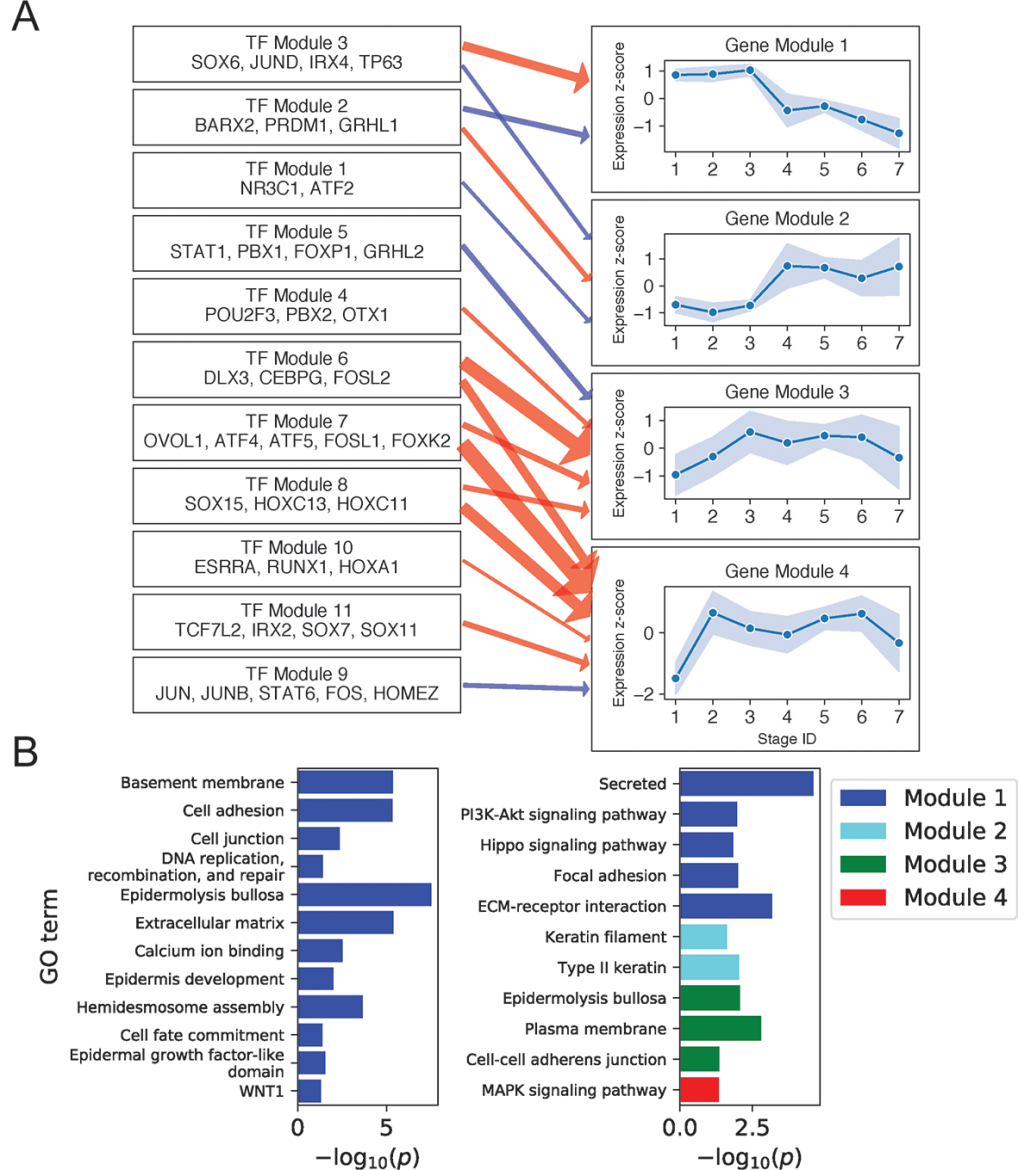
Basal keratinocyte network analysis identifies gene and TF modules specific to basal functions. **(A)** Regulation of four GO-enriched gene modules by TF modules, represented as a directed graph. Gene module nodes show log-transformed stage-wise mean imputed expression normalized across stages 1 to 7 with shading of 1 standard deviation interval. Transcription factor modules list their TF constituents. Arrows indicate regulation with width proportional to predicted strength of activation (red) or inhibition (blue). **(B)** Minus log of adjusted *p* values for selected GO terms enriched in each gene module. See also **Supplementary File 1: Figure S5**.

**Figure 3A** shows regulatory relationships for four gene modules enriched in gene ontology (GO) terms (**Figure 3B**) (see **Supplementary File 2: Table S4** for full GO output). Gene Module 1 was highly expressed in all BK stages and contained genes important for anchoring cells to the basement membrane and extracellular matrix *via* hemidesmosomes and other cell junctions, genes encoding extracellular signaling molecules, and genes participating in the key Hippo and PI3K intracellular signaling pathways. Transcription factors predicted to activate Module 1 genes recapitulated several established and independently predicted regulatory relationships. For example, TP63 and JUND are known to positively regulate *ITGB4* and *LAMA3A*, respectively (Virolle et al., 1998; Carroll et al., 2006), whereas IRX4 and JUND are both predicted regulators of hemidesmosome assembly (Lachmann et al., 2018).

Notably, four of the six genes in the Hippo pathway (*AJUBA*, *WNT7A*, *WNT7B*, and *WNT3*) and seven of the eight genes in the PI3K pathway (*ITGA3*, *LAMB4*, *LAMB3*, *FGFR2*, *COL4A6*, *ITGB4*, and *LAMA3*) were expressed as extracellular or cell membrane–associated proteins. Given that these pathways involve signaling *via* intracellular posttranslation modification, this result suggested that the primary mechanism for pathway modulation at the transcriptional level might be *via* changing the expression of extracellular signaling molecules and the cell membrane proteins that transduce these signals. Examining the position of Module 1 genes in the Hippo signaling pathway (Kanehisa et al., 2017) illustrated this mechanism and showed that Module 1 genes promoted the pro-proliferative Hippo-OFF signaling state (**Supplementary File 1: Figure S6**). Specifically, the Module 1 cell membrane–associated protein AJUBA and intracellular protein RASSF6 are known to repress MST1/2, allowing nuclear localization of YAP/TAZ, which defines the pro-proliferative Hippo-OFF state (Meng et al., 2016). In the nucleus, TFs activated downstream of Module 1 extracellular WNT signaling proteins (WNT7A, WNT7B, and WNT3) can interact with YAP to promote pro-proliferative genes, including the Module 1 gene *CCND2* (Kanehisa et al., 2017).

Module 2 genes were enriched for keratins and rose sharply in expression at stage 4. Consistent with the strong mitotic signal at this stage, two of the three keratins in this module (*KRT6A* and *KRT6B*) were previously implicated in rapid keratinocyte division (Bolognia et al., 2017). Moreover, KRT6A and KRT6B were also shown to suppress keratinocyte migration during wound repair (Rotty and Coulombe, 2012), suggesting that the sharp rise in *KRT6A/B* expression in stage 4 and its fall beyond stage 5 could help inhibit migration of this mitotic cell population from the basal layer (**Supplementary File 1: Figure S7**). The proposed mechanism of impaired migration may explain how this mitotic population remains in or near the basal layer, despite expressing spinous layer markers (e.g., KRT1 and KRT10) at higher levels than BK cells (**Supplementary File 1: Figure S3**).

Previous publications confirmed the function of several transcriptional regulators predicted for Gene Module 2. For example, TP63 knockdown was shown to increase the expression of *KRT6A* in human keratinocyte cell lines (Barbieri et al., 2006). Similarly, conditional knockout of glucocorticoid receptor NR3C1 in mouse keratinocytes was shown to increase the expression of *KRT6A*, *KRT6B*, and *KRT77*, another keratin in the Gene Module (Sevilla et al., 2013).

Gene Module 4 was enriched for MAPK signaling genes (*CRKL*, *FGF11*, *GADD45A*, *FLNB*, *DUSP7*, and *MYC*) and rose sharply in expression at stage 2. The overall effect of Module 4 gene expression on MAPK signaling was complex, with FGF11 and GADD45A activating the ERK and JNK pathways (Kanehisa et al., 2017); DUSP7 inhibiting ERK, JNK, and p38 pathways (Amit et al., 2007; Kanehisa et al., 2017); and CRKL and FLNB serving structural functions. Moreover, different outcomes have been reported for activation of MAPK signaling by Module 4 genes. On the one hand, activation of JNK and P38 pathways by the DNA damage response gene *GADD45A* can promote apoptosis and cell cycle arrest (Hildesheim et al., 2002). On the other hand, activation of ERK signaling by growth factor FGF11 may promote proliferation (Kim et al., 2008). These results, together with our finding of Gene Module 4 regulation by multiple TF modules, including MAPK regulatory targets FOS, JUN (Amit et al., 2007), and FOSL1 (Gillies et al., 2017), suggested complex regulation with multiple feedback mechanisms in controlling proliferation, differentiation, and apoptosis.

### Gene Modules in the Differentiated Network Promote Keratinization, Barrier Formation, and Down-Regulation of Basal State Signaling

**Figure 4:**
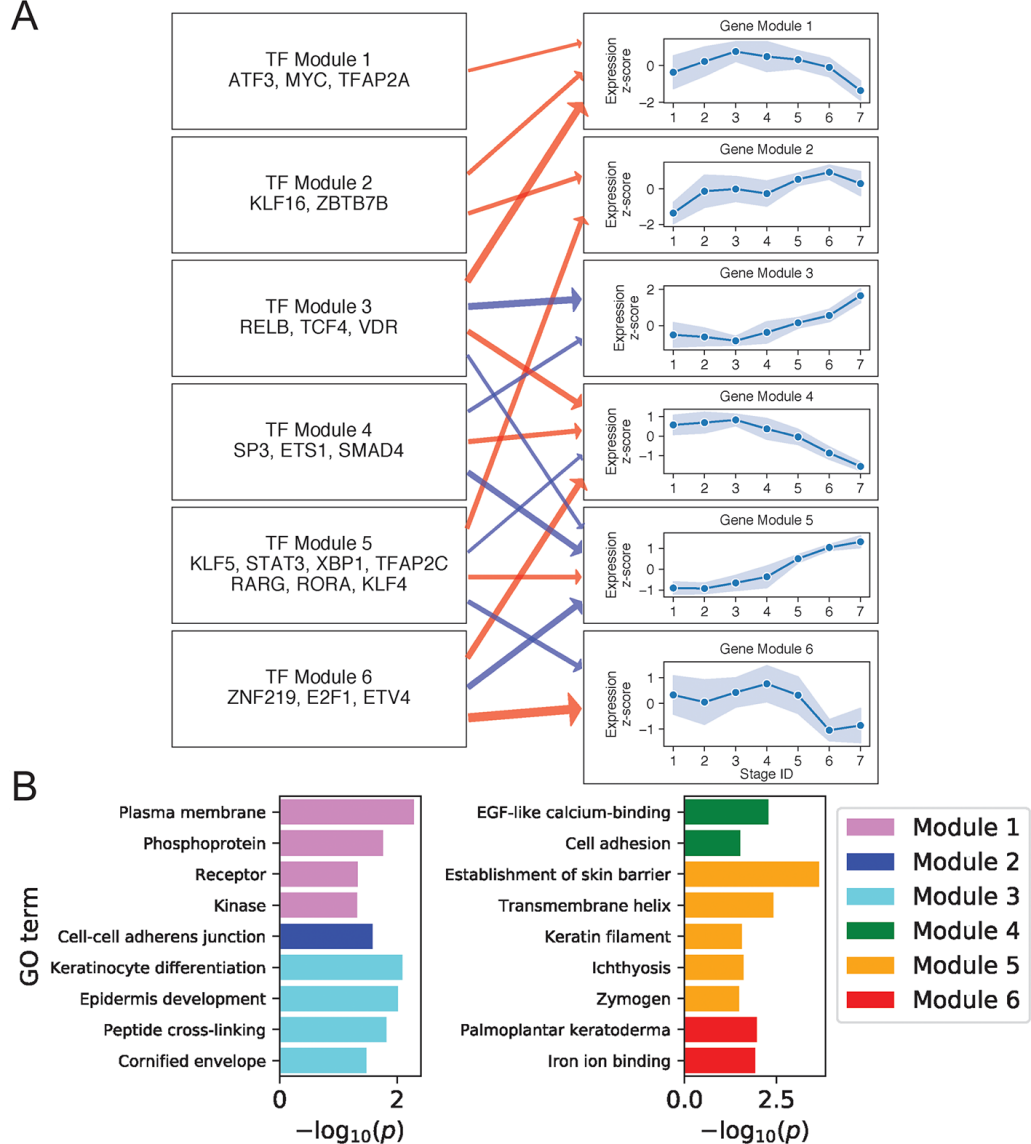
Differentiated keratinocyte network analysis identifies gene and TF modules specific to differentiated functions. **(A)** Regulation of six GO-enriched gene modules by TF modules, represented as a directed graph. Gene module nodes show log-transformed mean stage-wise imputed expression normalized across stages 1 to 7 with shading of 1 standard deviation interval. Transcription factor modules list their TF constituents. Arrows indicate regulation with width proportional to predicted strength of activation (red) or inhibition (blue). **(B)** Minus log of adjusted *p* values for selected GO terms enriched in each gene module. See also **Supplementary File 1: Figure S8**.

We next constructed regulatory relationships among gene and TF modules for the DK state using the same method described above, calculating gene correlations across cells in stages 4 to 7 and restricting attention to TFs with motifs enriched in DK-specific SEs (Methods). This analysis identified activating and inhibiting relationships among 21 gene and 9 TF modules (**Supplementary File 1: Figure S8**; **Supplementary File 2: Table S3**). **Figure 4A** shows regulatory relationships for six gene modules enriched in GO terms (**Figure 4B**) (see **Supplementary File 2: Table S4** for full GO output).

Gene Module 1 decreased in expression with differentiation and was enriched for GO terms associated with intercellular signal receptors and intracellular signaling cascades. Many Module genes associated with these terms were also seen to function in basal state signaling pathways. For example, Module genes in the Hippo pathway included cell membrane–associated *AJUBA*, *WNT7B*, and *DLG5* (Elbediwy et al., 2016; Kwan et al., 2016; Kanehisa et al., 2017). Module genes in the MAPK pathway included receptor tyrosine kinases *FGFR3* and *DDR1* (Hilton et al., 2008; Duperret et al., 2014), the kinases *MAPKBP1* and *TNK1* (Hoare et al., 2008; Lecat et al., 2012), the receptor *ADIPOR1* (Shibata et al., 2012), and the phosphoprotein and TF *ATF5*. The decreasing expression of this signaling module thus reflected a shift in the primary cellular function upon differentiation, with basal cells balancing self-renewal and amplification *via* abundant signaling between and within cells, while differentiated cells began suppressing signaling proteins in favor of those needed for barrier function. Several positive regulators of this Module are known to promote cell cycling, making them plausible regulators of the associated MAPK and Hippo pathways. These regulators included KLF16, which suppresses cyclin-dependent kinase inhibitor CDKN1A (Sakaguchi et al., 2005), and MYC, whose knockdown prevents keratinocyte proliferation (Wu et al., 2012).

Gene Module 4 also decreased with differentiation and was enriched for genes involved in EGF-like calcium binding and cell adhesion. Cell adhesion genes included several members of the cadherin superfamily: *CDH3*, *FAT1*, and *DSG3*. Predicted activators of this Module included our experimentally validated TF ETV4 (**Figure 2B**), which was previously shown to positively regulate cadherins in mouse spinal cord motor neurons, promoting segregation of cells with similar function (Livet et al., 2002; Helmbacher, 2018). Moreover, it was also demonstrated that ETV4 can positively regulate *RUNX1*, another Module 4 gene (Helmbacher, 2018). These findings thus supported that the cadherin regulatory function of ETV4 in the neuronal lineage may extend to keratinocytes.

Gene Module 3 increased its expression with differentiation and was enriched for genes related to the formation of cornified envelope and DK function. For example, the protein products of *LOR*, *SPRR1B*, and *CSTA* in this module are peptides cross-linked in the cornified envelope, while the keratinocyte differentiation protein ACER1 hydrolyzes ceramides, abundant in the granular layer, producing free sphingoid bases with antimicrobial function (Houben et al., 2006). Two other important epidermis development genes in this module were *KLK7* and *CALML5*; KLK7 degrades cellular adhesions of the cornified layer, favoring desquamation (Caubet et al., 2004), and CALML5 is thought to regulate differentiation by mediating cytoplasmic sequestration of YAP1 and initiating the antiproliferative Hippo-ON state (Sun et al., 2015). This gene module did not have positive TF regulators in our network, but had two sets of negative regulators (Modules 3 and 4). Of note, TF Module 4 contained SP3, ETS1, and SMAD4 that were previously shown to interact physically and suppress hematopoiesis (Morikawa et al., 2013; Raz et al., 2014). Our analysis thus indicated that steady reduction of these TFs contributed to the de-repression of Module 3 genes during differentiation.

Gene Module 5, like Module 3, increased its expression with differentiation and was negatively regulated by TF Modules 3 and 4. It contained genes primarily involved in barrier function, with several of these genes (*DEGS2*, *CERS3*, *ABCA12*, *TMEM79*) functioning in lipid synthesis and transport *via* the lamellar granule system. Other module members were involved in cell-cell adhesion (desmosomal proteins DSC1, DSG1, and PERP), tight junctions (CLDN1 and CLDN8), and desquamation (serine-proteases KLK8, KLK11) (Kishibe et al., 2007). Finally, the module also contained the enzymes TGM3 and CASP14 that promote cornification, DK-specific signaling molecules genes *KRTDAP* and *DMKN* (Matsui et al., 2004; Tsuchida et al., 2004), and the antimicrobial gene *DEFB1* (Ali et al., 2001). Apart from negative regulation by TF Modules 3 and 4, Gene Module 5 was positively regulated by TF Module 5. This TF module includes RORA, which is known to positively regulate *ABCA12* and other genes functioning in the granular lipid barrier (Dai et al., 2013). Our analysis thus identified Modules 3 and 5 genes as key components of keratinocyte terminal differentiation coordinately regulated by TFs that may preferentially localize in DK-specific SEs to either suppress or promote terminal differentiation.

### Antioxidant Gene Expression Is Enriched in the Basal State and Coupled to the Spatial Organization of Epidermis

**Figure 5:**
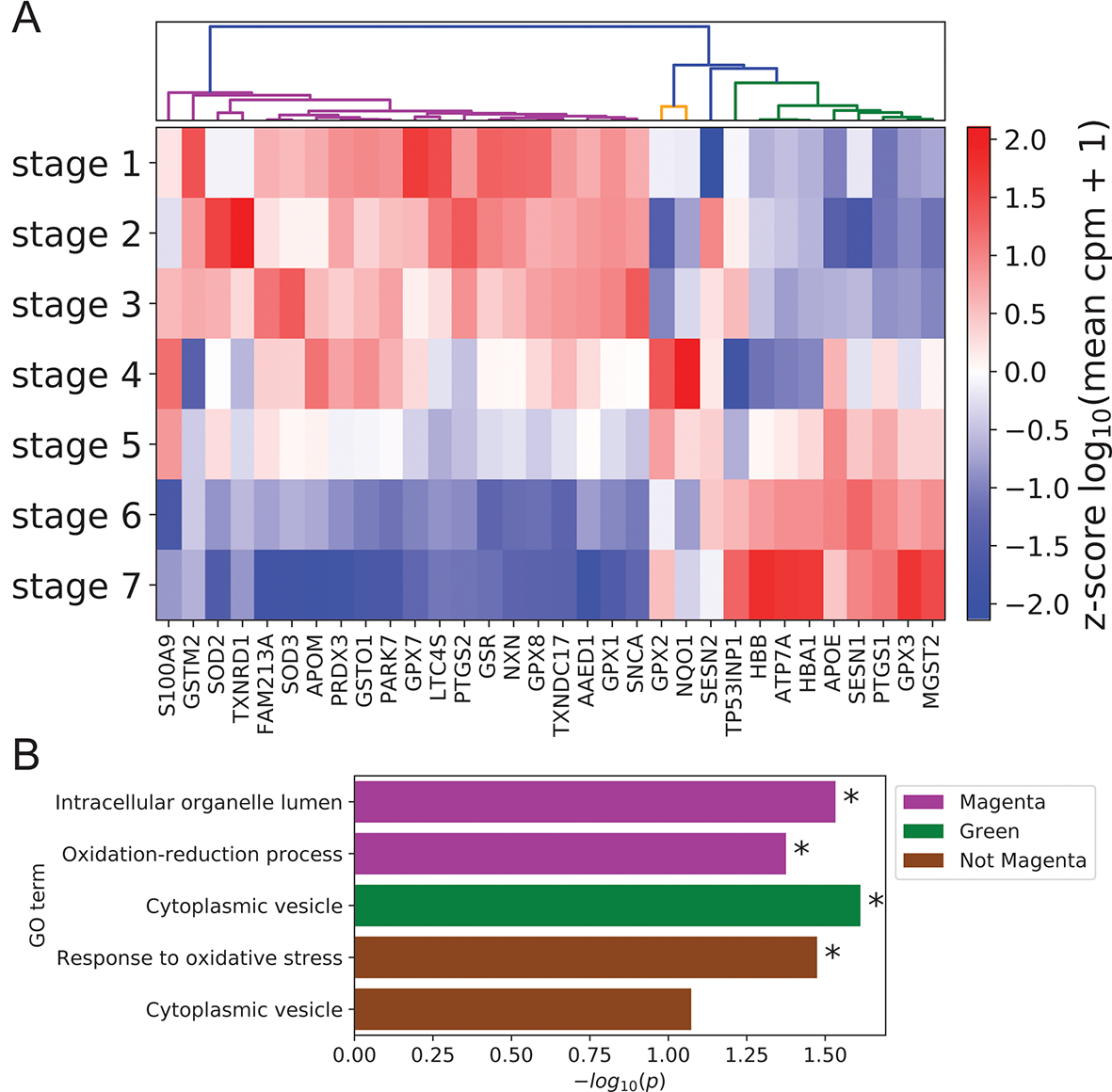
Peak expression of dynamic antioxidant genes is enriched in the BK state. **(A)** Log-transformed stage-wise mean imputed expression of dynamic antioxidant genes normalized across stages. Columns are organized by hierarchical clustering (Methods). **(B)** Minus log of unadjusted *p* values (Methods) for selected GO terms enriched in selected gene sets clustered from **(A)**. Asterisks indicate significance at 0.05 threshold.

Given the documented role of ROS and antioxidants in modulating keratinocyte differentiation (Hamanaka et al., 2013; Bhaduri et al., 2015), we also used our scRNA-seq data to examine coordination between antioxidant gene expression and differentiation state. Clustering of annotated antioxidant genes (Carbon et al., 2009) selected for dynamic expression across stages identified three distinct expression clusters (**Figure 5A**, Methods). The majority of antioxidant genes (20 of 32) belonged to the magenta cluster with peak expression in the basal state. The size of this cluster was significantly larger than expected by chance (*p* = 8.5 × 10^-4^, Methods), suggesting that antioxidant genes were preferentially expressed in the basal state to preserve self-renewal capacity by preventing ROS accumulation (Bigarella et al., 2014). In support of this conclusion, the magenta cluster contained the gene *SOD2* whose conditional knockout in mouse keratinocytes has been shown to induce cellular senescence and elevate the expression of differentiation marker genes at wound sites (Velarde et al., 2015).

The remaining two clusters (orange and green) attained peak expression in stages 4 to 5 and stages 5 to 7, respectively. Given the putative role of magenta class genes in preserving the basal state, we sought to identify distinct functions for these late peaking clusters. Gene ontology analysis revealed that magenta cluster proteins were enriched in organelle lumens; by contrast, green cluster gene products were enriched in cytoplasmic vesicles, with a similar trend holding for the group of all genes not in the magenta cluster (**Figure 5B**; **Supplementary File 2: Table S5**). This difference in cellular localization reflected potential differences in function, with magenta cluster proteins localized in key organelles to prevent the initiation of differentiation and green cluster proteins diffused throughout the cytoplasm to mitigate environmental oxidative stress and protect basal cells. Supporting this interpretation, the genes not in the magenta cluster were enriched for the GO term “response to oxidative stress” (**Figure 5B**).

## Discussion

Keratinocyte function in the basal and differentiated states depends on complex transcriptional regulation involving TFs, epigenetic modifications, and environmental queues from ROS levels and other stimuli. In this work, we have integrated bulk epigenetic profiles and single-cell expression data to better understand the coordination of these regulatory mechanisms. In particular, by considering known and predicted keratinocyte-specific TFs, we have uncovered that the turnover of this master set of TFs upon differentiation is coupled to the reported transition from BK to DK SEs. We have confirmed that synthetically suppressing the TFs ZBED2 and ETV4, identified in this work as crucial promoters of the basal state, leads to acute differentiation of BKs. We have also prioritized candidate promotors of differentiation that may be studied in subsequent experiments.

The single-cell transcriptomic data have also allowed us to identify a population of mitotic cells containing sharp expression spikes for established keratinocyte epigenetic regulators EZH2, DNMT1, and UHRF1, as well as for the enhancer-associated histone H2A.Z and the SWR1 remodeling complex that deposits the histone variant. The fact that *EZH2*, *DNMT1*, and *UHRF* peak expression coincides with the temporal stage of TF and SE turnover underscores the importance of these genes and helps localize their activity during differentiation pseudotime. Moreover, the co-occurrence of *H2A.Z* and SWR1 complex hypertranscription with this turnover suggests that these genes may have a previously unappreciated role in epigenetic regulation of keratinocyte transition from BK to DK states.

Network analysis has shown that TFs with differential binding in BK versus DK SEs regulate distinct sets of gene modules enriched for important keratinocyte functions. Consistent with previous studies, our BK network analysis has highlighted the role of TP63 in basement membrane adhesion and regulation of intercellular signaling pathways including WNT (Wu et al., 2012), as well as the importance of Hippo signaling in BKs (Elbediwy et al., 2016). Meanwhile, our DK analysis has identified regulators of terminal differentiation gene modules and implicated ETV4 in regulating cadherin superfamily genes, in a manner similar to its established function in motor neurons of the spinal cord (Livet et al., 2002; Helmbacher, 2018). The role of spinal cord cadherins in segregating cells by function suggests that a subset of ETV4 targets may also mediate epidermal cell sorting to assign specific keratinocyte functions to each epidermal layer.

As a proxy for measuring the degree of ROS suppression at each keratinocyte stage, we have demonstrated preferential expression of antioxidant genes in the BK state and uncovered differences in patterns of subcellular localization between BK- and DK-specific antioxidant genes. Notably, BK-specific antioxidant proteins tend to preferentially localize in organelles, such as the mitochondria, where they may control redox levels or the transduction of redox signals, preventing the onset of differentiation. This finding complements previous results that increased expression of select proteins localizing to the mitochondria promotes differentiation by increasing ROS levels (Bhaduri et al., 2015). By contrast, DK-specific antioxidant proteins tend to localize in cytoplasmic vesicles where they may be more important for epidermal barrier function than for regulation of differentiation.

Our integrative models of transcriptional regulation have shown that keratinocyte cell fate determination requires coordinating the expression level of critical TFs with the availability of their binding motifs in differentiation state-specific SEs. The inferred regulatory networks have provided insights into the transcriptional regulation of key genes essential for skin homeostasis and function. We have thus demonstrated that computational analyses of single-cell transcriptomic profiles in the context of other genomic and epigenomic data provide a powerful method for reconstructing cellular differentiation processes.

## Materials and Methods

### Keratinocyte Isolation and Primary Culture

Primary human keratinocytes were isolated from neonatal foreskin surgical tissue discards obtained with written informed consent using protocols approved by the UCSF institutional review board (#10-00944). Following the method of Lowdon et al. (2014), skin was incubated overnight at 4°C in 25 U/ml dispase solution (Corning Life Sciences, Corning, NY). Next, epidermis was mechanically separated from the dermis and incubated in 0.05% trypsin for 15 min at 37°C. Dissociated epidermal cells were filtered with a 100 μm nylon cell strainer (Corning Life Sciences) and then cultured in keratinocyte growth media (KGM; medium 154CF supplemented with 0.07 mM CaCl_2_ and Human Keratinocyte Growth Supplement; Life Technologies, Waltham, MA).

### Data Accession and Cell Selection

Raw counts of scRNA-seq data used in this study were obtained from the European Genome-phenome Archive (EGAS00001002927). The data were generated using Chromium Single Cell 3′ v2 libraries (10X genomics) from three human epidermal samples collected at each of four anatomical locations/disease conditions. Sequence demultiplexing resulted in counts of unique molecular identifiers (UMIs) for genes and noncoding RNA in more than 100,000 cells [see Cheng et al. (2018) for details]. Cell filtering and identification of keratinocytes followed Cheng et al.(2018), with 92,889 passing quality control metrics and 85,345 of these identified as keratinocytes based on average marker gene expression in published cell clusters. This manuscript mainly focuses on the foreskin data from this data set.

### RNAi Knockdown of Predicted TFs

ON-TARGETplus siRNA pools targeting *ETV4*, *ZBED2*, *BNC1*, and *HOXC11* as well as the ON-TARGETplus Nontargeting Control siRNA #1 were obtained from Dharmacon (Lafayette, CO). Pooled keratinocytes from five different individuals were seeded at a density of 300,000 cells/ml in 12-well plates. Within 30 min of plating, 10 nM siRNA plus 5 µL/well of Hiperfect transfection reagent (Qiagen, Germantown, MD) was added. Transfections were done in quadruplicates. At 48 hours after transfection, siRNA media was removed and replaced with 1 ml fresh KGM (medium 154CF supplemented with 0.07 mM CaCl2 and Human Keratinocyte Growth Supplement; Life Technologies). Five days after transfection, total RNA was extracted using TRIzol reagent (Life Technologies) following the manufacturer’s protocol. cDNA was synthesized using the iScript cDNA Synthesis Kit (Bio-Rad, Hercules, CA) following the manufacturer’s protocol. Quantitative polymerase chain reaction was performed with POWER SYBR Green Complete Master Mix (Life Technologies) to measure the expression levels of the housekeeping gene *GUSB*, as well as *ETV4*, *ZBED2*, *BNC1*, *HOXC11*, *KRT10*, and *FLG*. Each sample was measured in triplicate on the Applied Biosystems StepOne System. Melting curves were manually inspected to confirm specificity. When applicable, the results are presented as mean ± standard deviation. Statistical analysis was conducted using GraphPad Prism v5.0f (La Jolla, CA). Student *t* test was used to compare two separate sets of independent and identically distributed samples with *p* < 0.05 considered as significant.

### Expression Level of Candidate TFs in Cell Culture

To assess concordance between Candidate TF’s differentiation-promoting scores calculated from epidermal scRNA-seq data (Results: Knockdown of ETV4 and ZBED2, predicted promoters of the BK state, induces differentiation; **Supplementary File 3**: Prioritization of knockdown targets; **Figure S4**) and changes in bulk RNA expression of these TFs during *in vitro* differentiation, we generated RNA-seq expression for primary cultured human keratinocytes cultured in basal/proliferating (0.07 mM Ca) or high calcium–induced differentiation (1.2 mM Ca) conditions. Negative control siRNA-treated keratinocytes were used a proxy for normal cultured keratinocytes. Keratinocytes were initially seeded at a density of 100,000 and 150,000 cells in 12-well plates using KGM with 0.07 mM Ca. Within 30 min of plating, 10 nM of either ON-TARGETplus Nontargeting Control siRNA #1 or 2 mixed with 2.5 µl/well of Hiperfect transfection reagent was added. At ∼48 h after transfection, subconfluent 100,000-cell wells were harvested using 0.5 ml TRIzol reagent (Life Technologies) for RNA extraction as per manufacturer’s protocol. At ∼48 h after transfection, the 150,000-cell wells had reached confluency, and the media was replaced with 1 ml fresh KGM with 1.2 mM Ca. After 24 h of exposure to high 1.2 mM calcium, the confluent cells were also harvested using 0.5 ml TRIzol reagent, and RNA-seq was performed. RNA-seq library preparation was performed using KAPA Biosystems Stranded RNA-Seq Kits and RiboErase HMR (Roche, Pleasanton, CA) with 300 to 1,000 ng of total RNA. To minimize batch effects, technical duplicate libraries were generated for each sample. Ribosomal RNA was depleted by hybridization of complementary DNA oligonucleotides plus treatment with RNase H and DNase to remove ribosomal RNA duplexed to DNA and original DNA oligonucleotides, respectively. RNA fragmentation was conducted using heat and magnesium. Using random primers, first-strand complementary DNA (cDNA) synthesis was conducted followed by second-strand synthesis, and A-tailing was added to the 3′ ends using dAMP. Fragments were amplified using appropriate adapter sequences *via* ligation-mediated polymerase chain reaction. Then, the libraries were quantitated with either Quant-iT dsDNA or Qubit dsDNA HS assay kits (Life Technologies). Quality assessment was performed using the LabChip GX Touch HT microfluidics platform (Perkin Elmer, Waltham, MA). 2 × 150 base pair sequencing on a NovaSeq 6000 instrument was performed on libraries with a PhiX Control v3 (Illumina, San Diego, CA). The RNA-Seq by Expectation Maximization algorithm (Li and Dewey, 2011) was used to quantify gene expression in terms of FPKM for technical replicates in both biological conditions. Change in expression between the differentiation-promoting (1.2 mM Ca) and non–differentiation-promoting (0.07 mM Ca) conditions was quantified as the log_2_ ratio of gene expression averaged over technical replicates (Figure S4).

### Identification of Keratinocyte-Specific Genes and Transcription Factors

Our objective of uncovering regulators and regulatory mechanisms specific to the keratinocyte lineage prompted us to focus analysis on genes and TFs with increased expression in keratinocytes compared with other types of primary cells. On the one hand, focusing on keratinocyte-specific genes and TFs had two benefits: first, it permitted discovery of gene modules particular to keratinocyte functions; and, second, it reduced false positives in our identification of keratinocyte regulators from single-cell data by adding a filter for specificity of expression across primary cells. On the other hand, recognizing that some TFs known to be important for keratinocyte regulation may also function in other cell types, we supplemented the data-driven identification of Keratinocyte TFs with a set of established keratinocyte regulators from the literature.

Identification of genes and TFs with significantly increased expression in keratinocytes used the expression data from the FANTOM consortium (Fantom Consortium et al., 2014). Relative log expression–normalized expression values for transcription start sites identified from cap analysis of gene expression (CAGE) experiments were obtained from http://fantom.gsc.riken.jp/5/datafiles/latest/extra/CAGE_peaks/hg19.cage_peak_phase1and2combined_tpm_ann.osc.txt.gz. Restricting to 495 human primary cell samples not marked for exclusion from expression analysis in Table S2 of (Fantom Consortium et al., 2014), we computed gene-level expression values by associating with each gene’s EntrezID the sum of CAGE peak expression values annotated with that ID. We used the Mann-Whitney *U* test to identify genes and TFs differentially expressed in three keratinocyte samples relative to the remaining 491 samples (due to our interest in epidermal keratinocytes, we excluded the oral keratinocyte sample from consideration). A list of annotated TFs (Zhang et al., 2015) was used to distinguish TFs from other protein coding genes and noncoding RNA. Genes and TFs with Benjamini−Hochberg false discovery rate (FDR) less than 0.05 and increased average expression in keratinocytes were selected and filtered to include only those with at least 1 UMI (raw data) in at least 1% of all single-cell keratinocytes (**Supplementary File 2: Table S1**). This differential expression and filtering procedure yielded 793 genes, termed FANTOM genes, and 49 TFs.

The set of differentially expressed TFs, prior to filtering for minimum scRNA-seq expression level, contained several members of the HES superfamily: *HES2*, *HES5*, and *HES7*. Of these, only *HES2* passed the filter. However, we observed that two other superfamily members, *HES1* and *HES4*, were robustly expressed and possessed dynamic expression patterns across our single-cell data (**Figure 1**). For this reason and because HES genes are targets of Notch signaling that has an established function in keratinocyte differentiation (Watt et al., 2008), we elected to add *HES1* and *HES4* to the set of 49 TFs. Below, we refer to the full set of 51 TFs as FANTOM TFs. We supplemented our FANTOM TFs with additional 49 TFs previously shown to regulate keratinocyte differentiation (Klein et al., 2017). Lowly expressed TF were filtered using the threshold on single-cell expression as described above. We refer to this set as Klein TFs.

From these FANTOM genes, FANTOM TFs, and Klein TFs, we constructed the final three sets for further analysis. The set termed Keratinocyte TFs consisted of the union of FANTOM TFs and Klein TFs and was used to study the dynamics of TF expression across single-cell stages, as well as for regulatory network analysis. The set termed Candidate Keratinocyte TFs consisted of FANTOM TFs not in the set of Klein TFs and was the focus of TF prioritization and validation. Finally, the set termed Keratinocyte Genes consisted of the union of Keratinocyte TFs and FANTOM genes and comprised the set of candidate target genes for regulatory network analysis. **Supplementary File 1: Figure S1** illustrates the construction of these sets, and **Supplementary File 2: Table S2** lists the sets’ genes.

### Summary of scRNA-Seq Data Processing and Analysis

Imputed gene expression was calculated as in Cheng et al. (2018). Briefly, we used the ZINB-WaVE algorithm (Risso et al., 2018) to obtain a low-dimensional, bias-corrected representation of raw singe-cell data, which were then used to construct a distance-based measure of cell similarly and perform imputation with the MAGIC algorithm (version 0.0) (van Dijk et al., 2018). Next, we selected foreskin keratinocytes based on their membership in expression-based clusters previously characterized as keratinocytes in (Cheng et al., 2018). We identified differentiation stages within this cell population by applying principal components analysis followed by k-means–based approximate spectral clustering (Yan et al., 2009) (**Supplementary File 3**: Identification of keratinocyte stages). To reduce false positives in downstream correlation analysis, we removed outlier cells from the eight keratinocyte stages identified by clustering, reduced MAGIC’s imputation time parameter, and reimputed (**Supplementary File 1: Figures S9–10**; **Supplementary File 2: Table S6**; **Supplementary File 3**: Calculation of gene correlations).

To construct **Figures 1** and **5**, Keratinocyte TFs and antioxidant genes were filtered for dynamic expression based on stage-wise log fold change and clustered using Pearson correlation distance among vectors of log-transformed stage-wise mean imputed counts per million (cpm) (**Supplementary File 3**: Clustering transcription factor expression trajectories and super-enhancer differential motif enrichment, antioxidant analysis). To prioritize Candidate Keratinocyte TFs for experimental validation, TFs were ranked by the sum of signed log-fold change of their target Keratinocyte Genes during differentiation (positive sign for activation, negative sign for repression). Targets were identified based on strength of TF-gene correlation/anticorrelation (**Supplementary File 3**: Prioritization of knockdown targets).

Regulatory analysis for the BK state used Keratinocyte TFs with motifs enriched in BK-specific SEs compared with DK-specific SEs and Keratinocyte Genes not down-regulated in the BK state compared with the DK state (Methods: Differential expression). Identification of gene and TF modules in the BK state used hierarchical clustering on signed expression similarity scores calculated as soft-thresholded Pearson correlation (Zhang and Horvath, 2005) of log-transformed imputed expression across cells in stages 1 to 4. We identified regulatory relationships between gene and TF modules by considering the distribution of magnitudes of mean similarity scores between all TF-gene module pairs:

$$\left\{\left|\underset{i\in A,j\in B}{\text{mean}}\;S_{i,j}\right|\text{ : }A\in \text{TF Modules, B}\in \text{Gene Modules}\right\}$$

where, following the notation of **Supplementary File 3**: Regulatory network construction, *s* *_i,j_* denotes the signed similarity score of TF *i* and target gene *j* (**Supplementary File 1: Figure S5B**). Regulatory relationships were assigned for module pairs exceeding the threshold illustrated in **Supplementary File 1: Figure S5(C, D)**. Regulatory analysis for the DK state used an analogous method [**Supplementary File 1: Figures S8(B–D)**]. Further details are given in **Supplementary File 3**: Regulatory network construction.

Source code used to generate results is available at https://github.com/jssong-lab/kcyteReg.

### Differential Expression

We used differential expression analysis to identify Keratinocyte Genes specific to the BK (union of stages 1, 2, 3) and DK (union of stages 5, 6, 7) states. First, log (cpm + 1) of nonimputed expression values was calculated for Keratinocyte Genes and for other genes with at least 3 UMIs in 20 foreskin keratinocytes. Next, we used limma-trend version 3.23.9 (Ritchie et al., 2015) to obtain moderated log_2_ fold-change values between the two states, as well as adjusted *p* values for differential expression tests (**Supplementary File 2: Table S7**). Finally, we defined Keratinocyte Genes specific to the BK versus DK states to be those genes differentially expressed at 5% FDR and with magnitudes of moderated log_2_ fold change greater than 0.25.

### Gene Ontology Analysis

We used the DAVID GO resource (Huang da et al., 2009) to determine functional enrichment in BK and DK gene modules, as well as in clusters of antioxidant genes with similar dynamic gene expression patterns. For BK and DK gene modules, we used the R library RDAVIDWebService (Fresno and Fernandez, 2013) to query DAVID with backgrounds composed of members of each gene module and a common control set of 12,516 expressed genes with at least 1 UMI in at least 1% of all keratinocytes. Bar plots in **Figures 3B** and **4B** show selected GO terms with Benjamini−Hochberg adjusted *p* < 0.05. **Supplementary File 2: Table S4** provides the full DAVID output for all gene modules identified for the BK and DK states. Gene ontology analysis for clusters of dynamically expressed antioxidant genes used the set of 65 antioxidants with at least 1 UMI in at least 1% of all keratinocytes (**Supplementary File 2: Table S2**). Because of the small sizes of gene sets and the large number of enrichment tests performed by DAVID, we did not find any significant enrichment after Benjamini-Hochberg correction for multiple hypothesis testing. We therefore reported uncorrected *p* values for selected GO terms in **Figure 5B**; **Supplementary File 2: Table S5** provides the full DAVID output.

## Data Availability

The data sets analyzed for this study can be found at the European Genome-phenome Archive (https://www.ebi.ac.uk/ega/home) (EGAS00001002927) (single-cell RNAseq data) and at the FANTOM consortium website (http://fantom.gsc.riken.jp/5/datafiles/latest/extra/CAGE_peaks/hg19.cage_peak_phase1​and2combined_tpm_ann.osc.txt.gz) (CAGE data). Genomic coordinates of super-enhancers characteristic of basal and differentiated keratinocytes were obtained from Klein et al. (2017). Source code used to generate results is available at https://github.com/jssong-lab/kcyteReg.

## Ethics Statement

Primary human keratinocytes were isolated from neonatal foreskin surgical tissue discards obtained with written informed consent using protocols approved by the UCSF institutional review board (#10-00944).

## Author Contributions

RC, JC, and JS conceived and supervised the project. AF carried out most of the computational analyses, aided by AL. PH and JL performed the validation experiments. AF, RC, JC, and JS wrote the manuscript with contributions from other authors. All authors read and approved the final manuscript.

## Funding

This work was supported in part by funds from NIH R01CA163336 and the Grainger Engineering Breakthroughs Initiative to JS, the L.S. Edelheit Family Biological Physics Fellowship to AF, and NIH K08AR067243 to JC.

## Conflict of Interest Statement

The authors declare that the research was conducted in the absence of any commercial or financial relationships that could be construed as a potential conflict of interest.

## Abbreviations

TF, transcription factor; BK, basal keratinocyte; DK, differentiated keratinocyte; SE, super-enhancer; ROS, reactive oxygen species; GO, gene ontology; cpm, counts per million; TSS, transcription start site; CAGE, cap analysis of gene expression.
