# Supplementary material for: Single-Cell Transcriptomics Reveals Spatial and Temporal Turnover of Keratinocyte Differentiation Regulators
Source: Front Genet. 2019 Sep 3;10:775. doi: 10.3389/fgene.2019.00775 (PMC6733986; doi:10.3389/fgene.2019.00775)
Supplement: Supplementary file 1 [file DataSheet_1.pdf]

Supplementary Figures

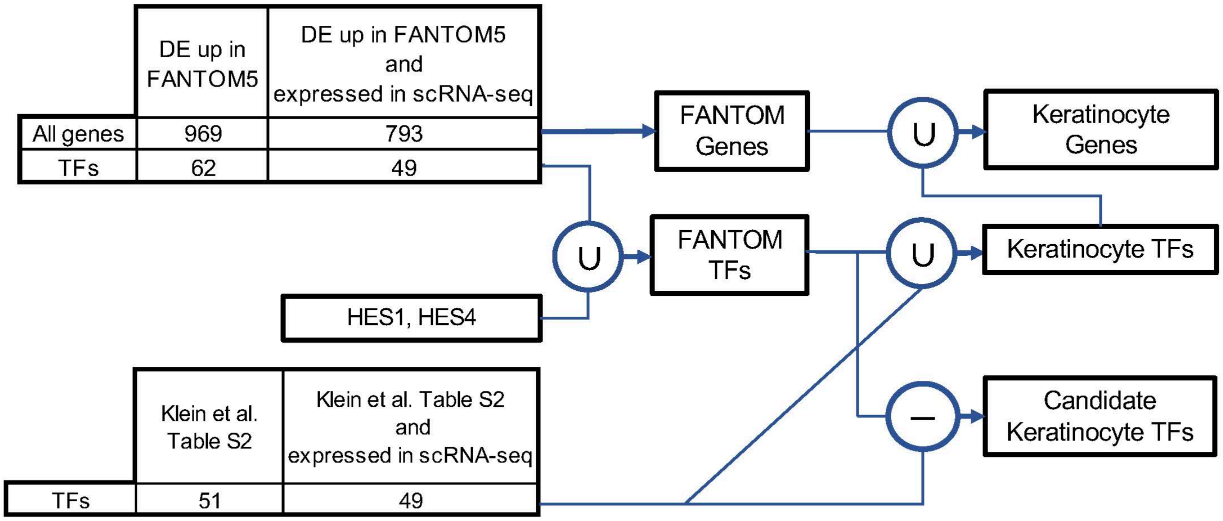

Figure S1: Flowchart for selection of TFs and target genes for investigation. Specificity of expression across human primary cells, manual curation and literature were used to construct three sets for downstream analysis: Keratinocyte TFs, Candidate Keratinocyte TFs and Keratinocyte Genes (see Methods: Identification of keratinocyte specific genes and transcription factors).

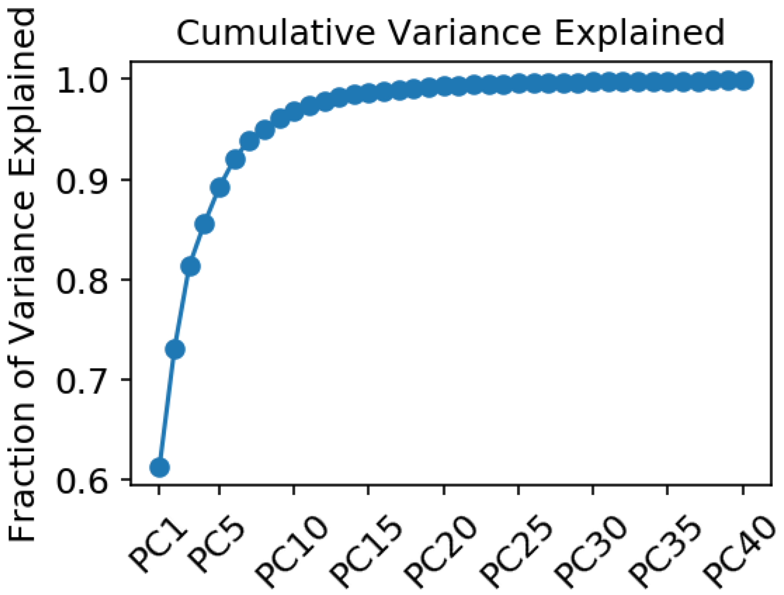

Figure S2: Cumulative fraction of variance in log transformed imputed expression explained vs principal component index

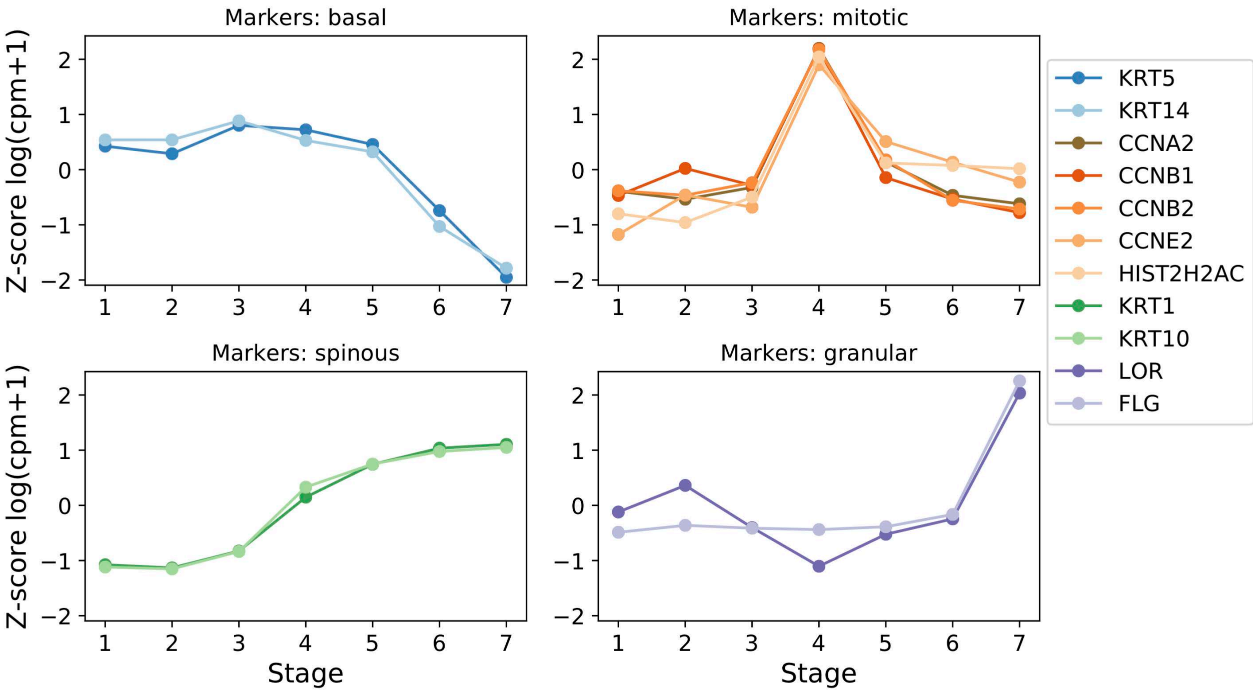

Figure S3: Log transformed stage-wise mean imputed expression (standardized across stages 1-7) of marker genes for the basal keratinocytes, mitotic activity, spinous keratinocytes and granular keratinocytes.

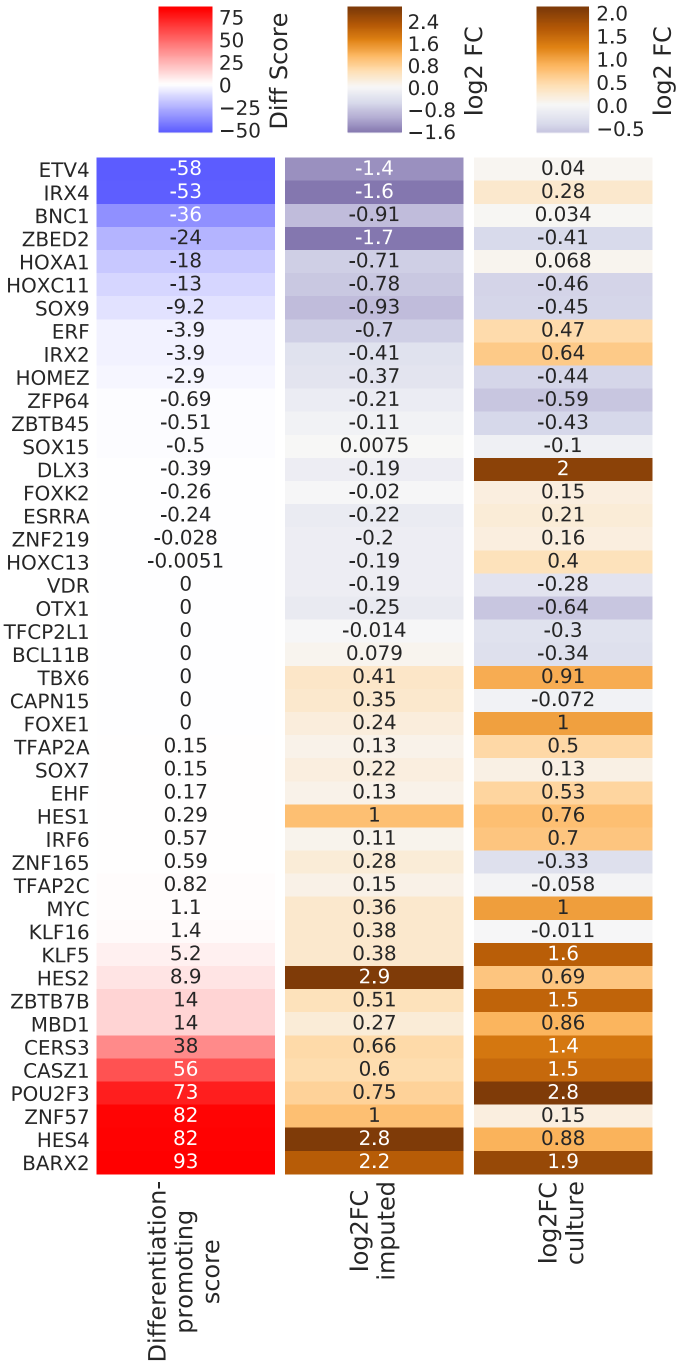

Figure S4: Statistics used to prioritize TFs for experimental validation. Differentiation score is assigned to each TF based on summed log-fold change of putative target genes, accounting for the sign of TF-target correlation (Supplementary Methods: Prioritization of knockdown targets). "Log2FC imputed" denotes log2 ratio of average single-cell imputed expression for DK state to average single-cell imputed expression for the BK state. "Log2FC culture" denotes log2 ratio of expression values (FPKM) for keratinocytes cultured in 1.2 mM Ca condition (differentiation promoting) to expression values for keratinocytes cultured in 0.07 mM Ca condition (non-differentiation promoting) (Methods: Expression level of Candidate TFs in cell culture).

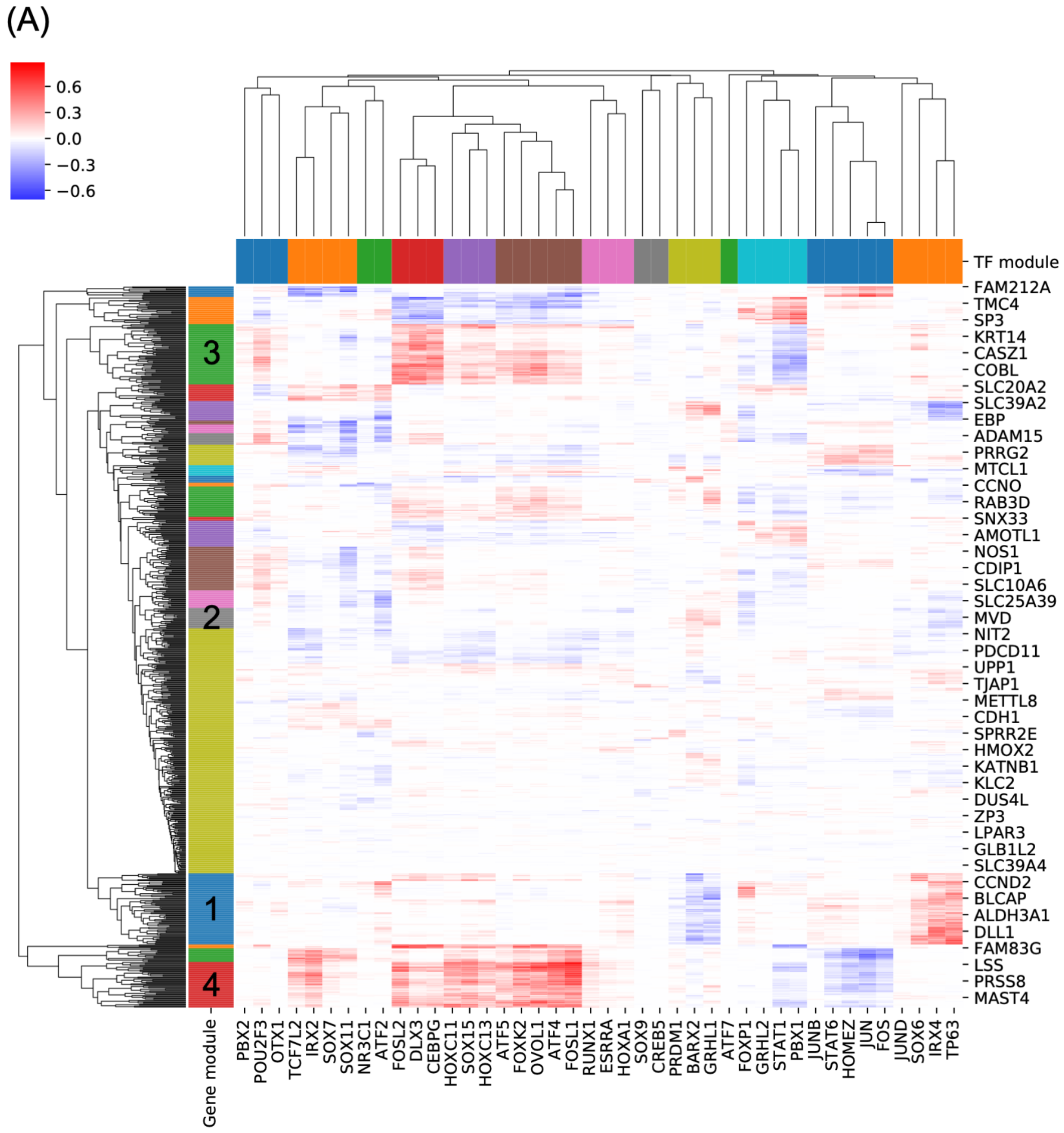
(A)
0.6
0.3
0.0
−0.3
−0.6
TF module
Gene module
3
2
1
4
FAM212A
TMC4
SP3
KRT14
CASZ1
COBL
SLC20A2
SLC39A2
EBP
ADAM15
PRRG2
MTCL1
CCNO
RAB3D
SNX33
AMOTL1
NOS1
CDIP1
SLC10A6
SLC25A39
MVD
NIT2
PDCD11
UPP1
TJAP1
METTL8
CDH1
SPRR2E
HMOX2
KATNB1
KLC2
DUS4L
ZP3
LPAR3
GLB1L2
SLC39A4
CCND2
BLCAP
ALDH3A1
DLL1
FAM83G
LSS
PRSS8
MAST4
PBX2
POU2F3
OTX1
TCF7L2
IRX2
SOX7
SOX11
NR3C1
ATF2
FOSL2
DLX3
CEBPG
HOXC11
SOX15
HOXC13
ATF5
FOXK2
OVOL1
ATF4
FOSL1
RUNX1
ESRRA
HOXA1
SOX9
CREB5
PRDM1
BARX2
GRHL1
ATF7
FOXP1
GRHL2
STAT1
PBX1
JUNB
STAT6
HOMEZ
JUN
FOS
JUND
SOX6
IRX4
TP63

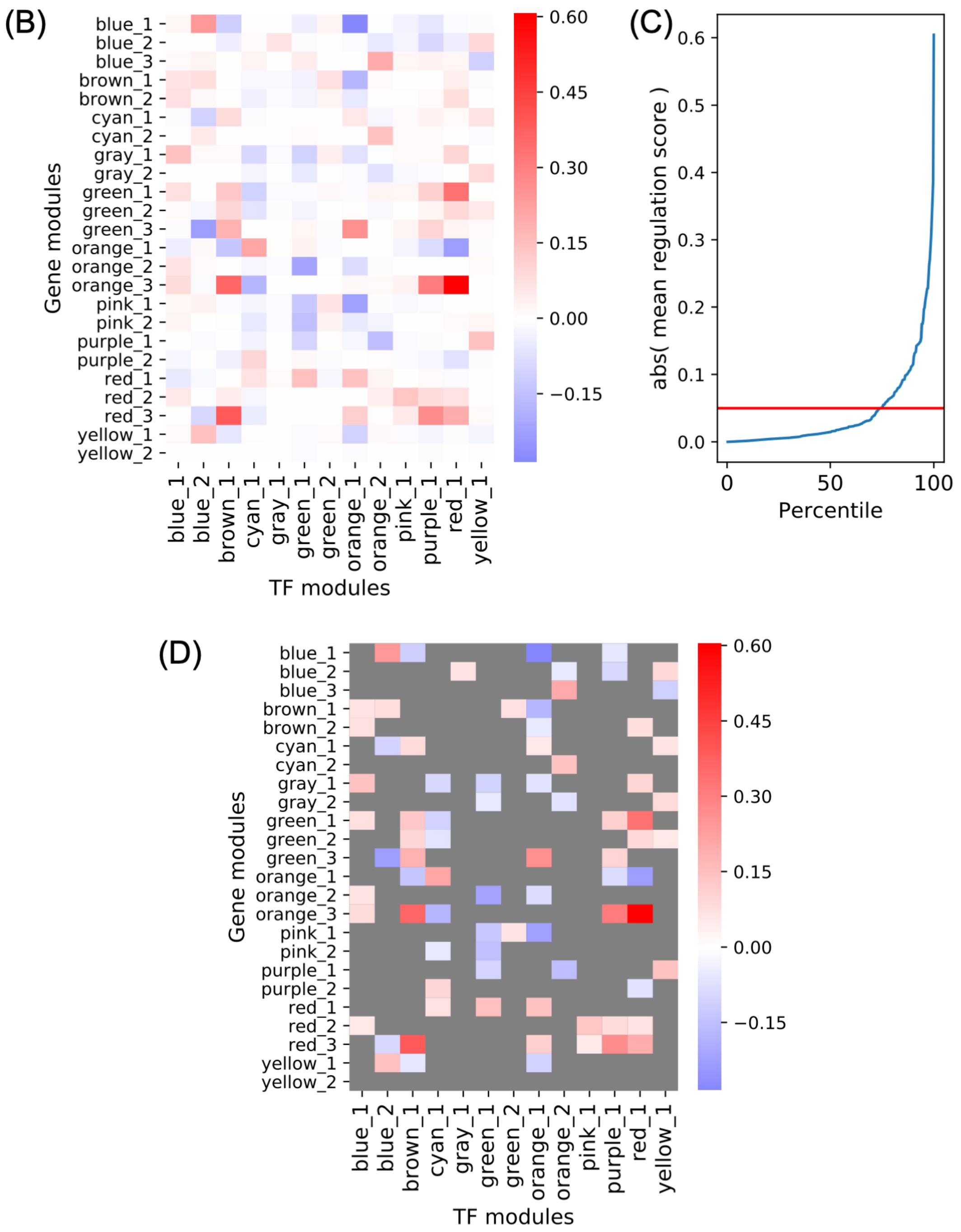

Figure S5: Graphical representation of BK network construction . (A) Heatmap of similarity scores between TFs (putative BK regulators) and target genes. Supplementary Methods: Regulatory Network Construction describes clustering of columns into TF modules (identified by colors in first row) and rows into gene modules identified by colors in first column. Numbers in first column correspond to gene modules in figure 3. (B) Heatmap of average TF, target gene similarity scores for pairs of TF and gene modules. (C) Inverse cumulative distribution of absolute value of average similarity scores in (B). Red horizontal line indicates threshold on magnitude of similarity score used to call TF-gene module regulatory relationships. (D) same as (B) but with average similarity scores not passing threshold masked in gray.

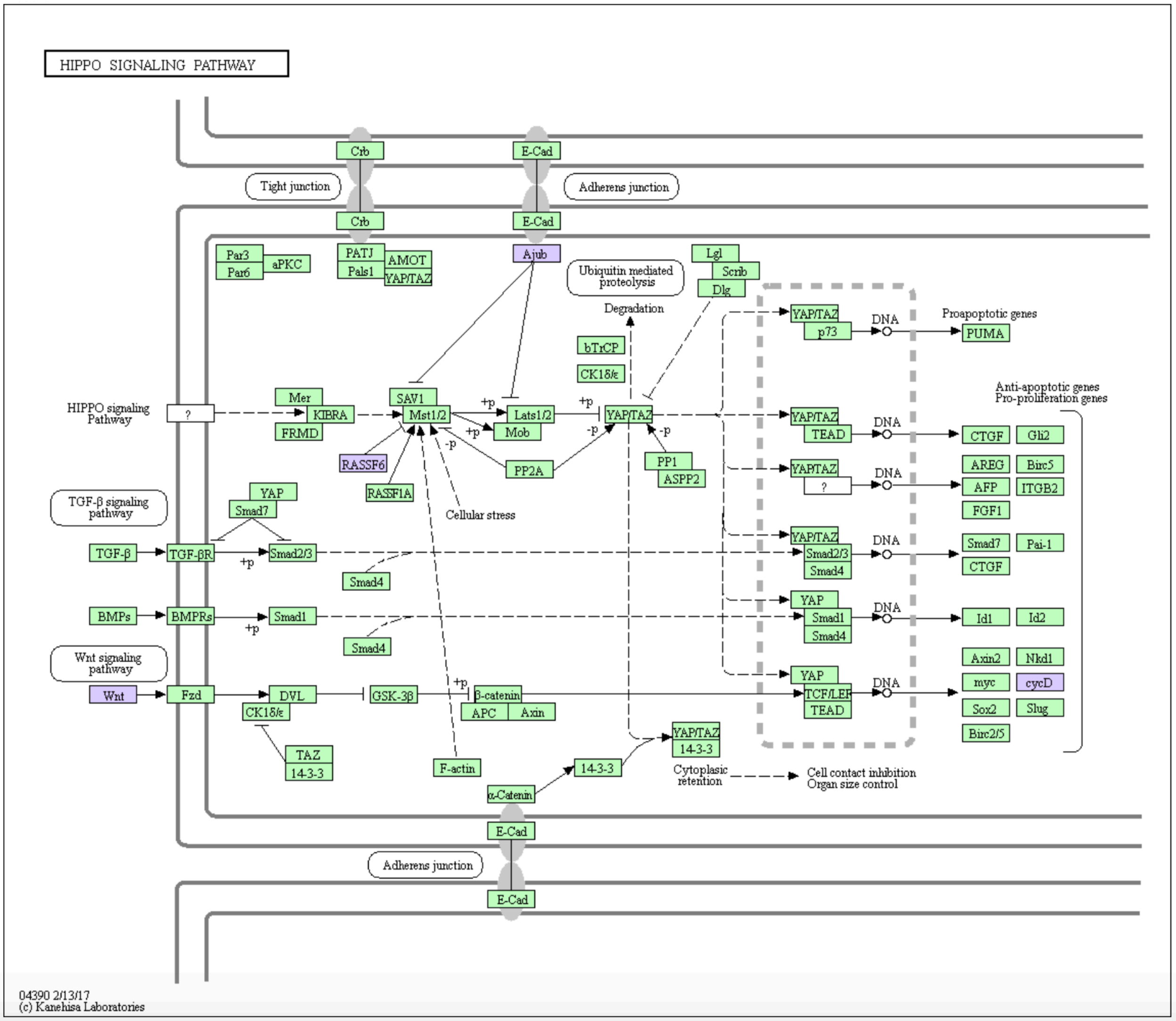

Figure S6: Genes corresponding to purple background proteins belong to BK network Gene Module 1. The net effect of AJUBA and RASSF6 is to inhibit phosphorylation of YAP thus allowing nuclear localization. Intracellular signaling downstream of WNT7A, WNT7B and WNT3 promotes activation of pro-proliferative genes by YAP and other TFs (Kanehisa et al., 2017).

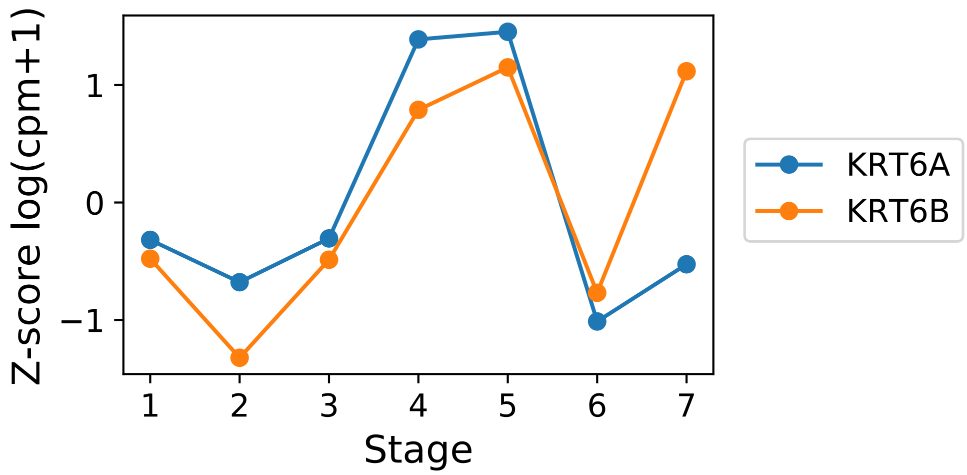

Figure S7: Log transformed stage-wise mean imputed expression (standardized across stages 1-7) of KRT6A and KRT6B.

(A)

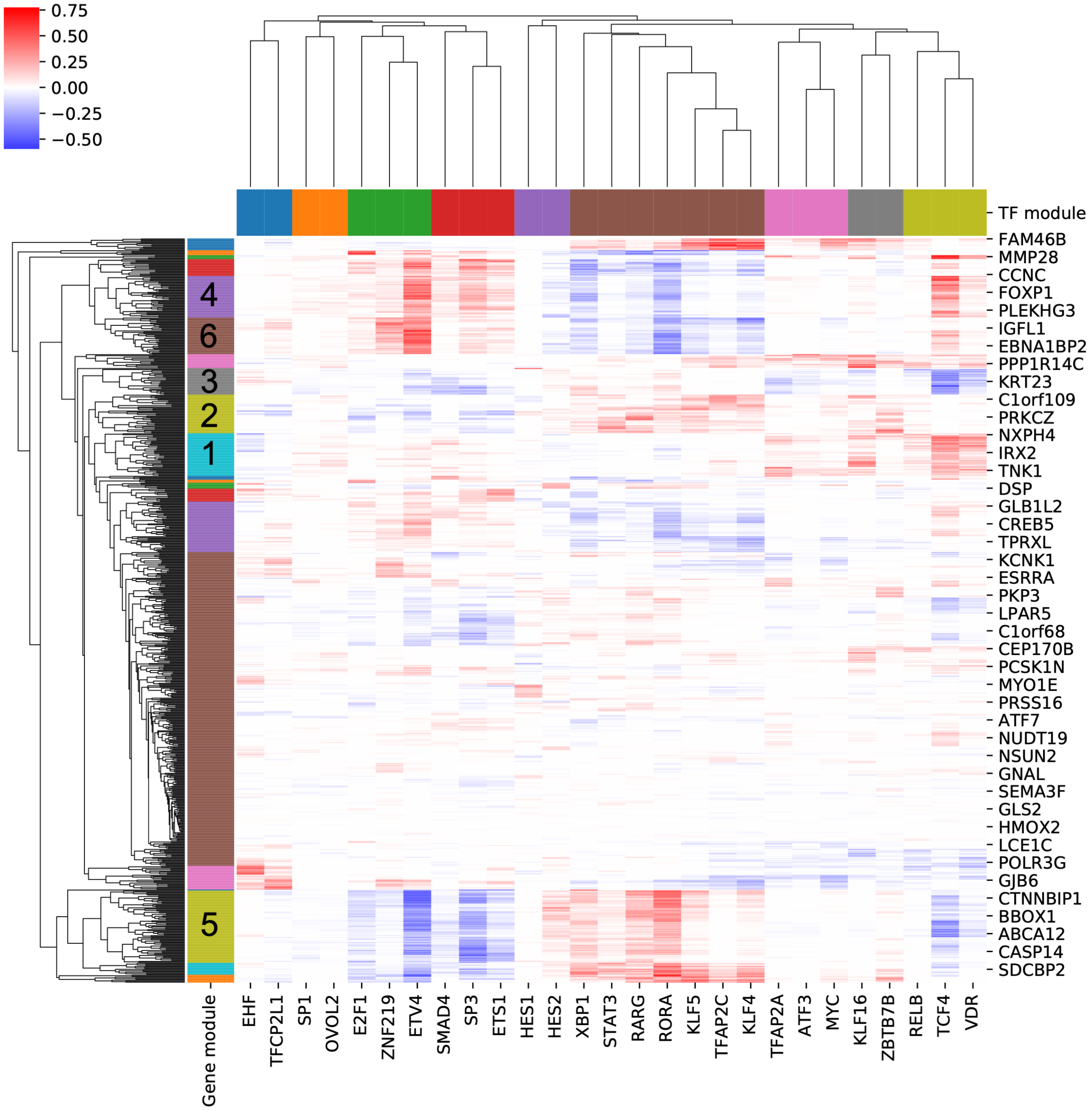
0.75
0.50
0.25
0.00
−0.25
−0.50
TF module
Gene module
4
6
3
2
1
5
FAM46B
MMP28
CCNC
FOXP1
PLEKHG3
IGFL1
EBNA1BP2
PPP1R14C
KRT23
C1orf109
PRKCZ
NXPH4
IRX2
TNK1
DSP
GLB1L2
CREB5
TPRXL
KCNK1
ESRRA
PKP3
LPAR5
C1orf68
CEP170B
PCSK1N
MYO1E
PRSS16
ATF7
NUDT19
NSUN2
GNAL
SEMA3F
GLS2
HMOX2
LCE1C
POLR3G
GJB6
CTNNBIP1
BBOX1
ABCA12
CASP14
SDCBP2
EHF
TFCP2L1
SP1
OVOL2
E2F1
ZNF219
ETV4
SMAD4
SP3
ETS1
HES1
HES2
XBP1
STAT3
RARG
RORA
KLF5
TFAP2C
KLF4
TFAP2A
ATF3
MYC
KLF16
ZBTB7B
RELB
TCF4
VDR

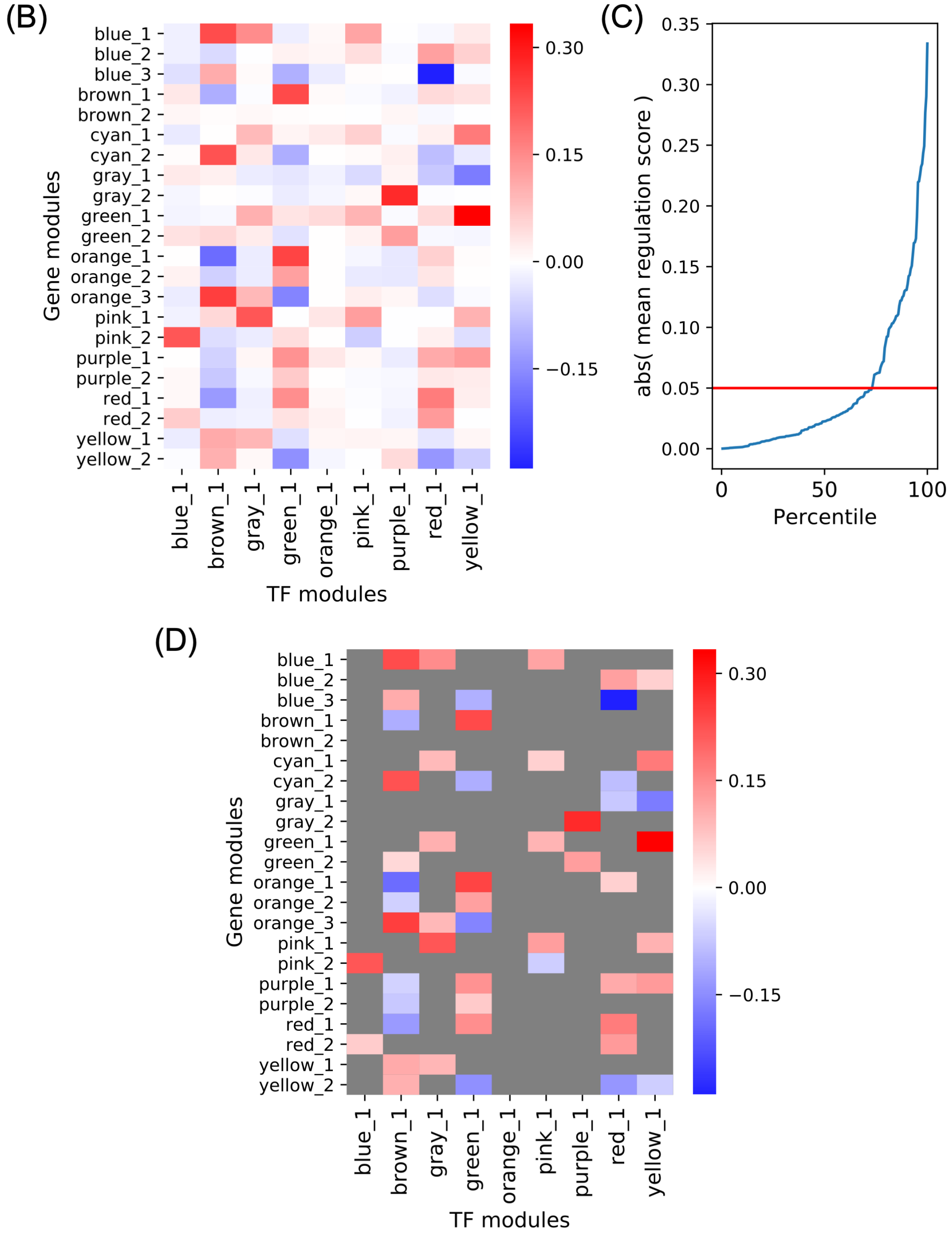

Figure S8: Graphical representation of DK network construction. (A) Heatmap of similarity scores between TFs (putative DK regulators) and target genes. Supplementary Methods: Regulatory Network Construction describes clustering of columns into TF modules (identified by colors in first row) and rows into gene modules identified by colors in first column. Numbers in first column correspond to gene modules in figure 4. (B) Heatmap of average TF, target gene similarity scores for pairs of TF and gene modules. (C) Inverse cumulative distribution of absolute value of average similarity scores in (B). Red horizontal line indicates threshold on magnitude of similarity score used to call TF-gene module regulatory relationships. (D) same as (B) but with average similarity scores not passing threshold masked in gray.

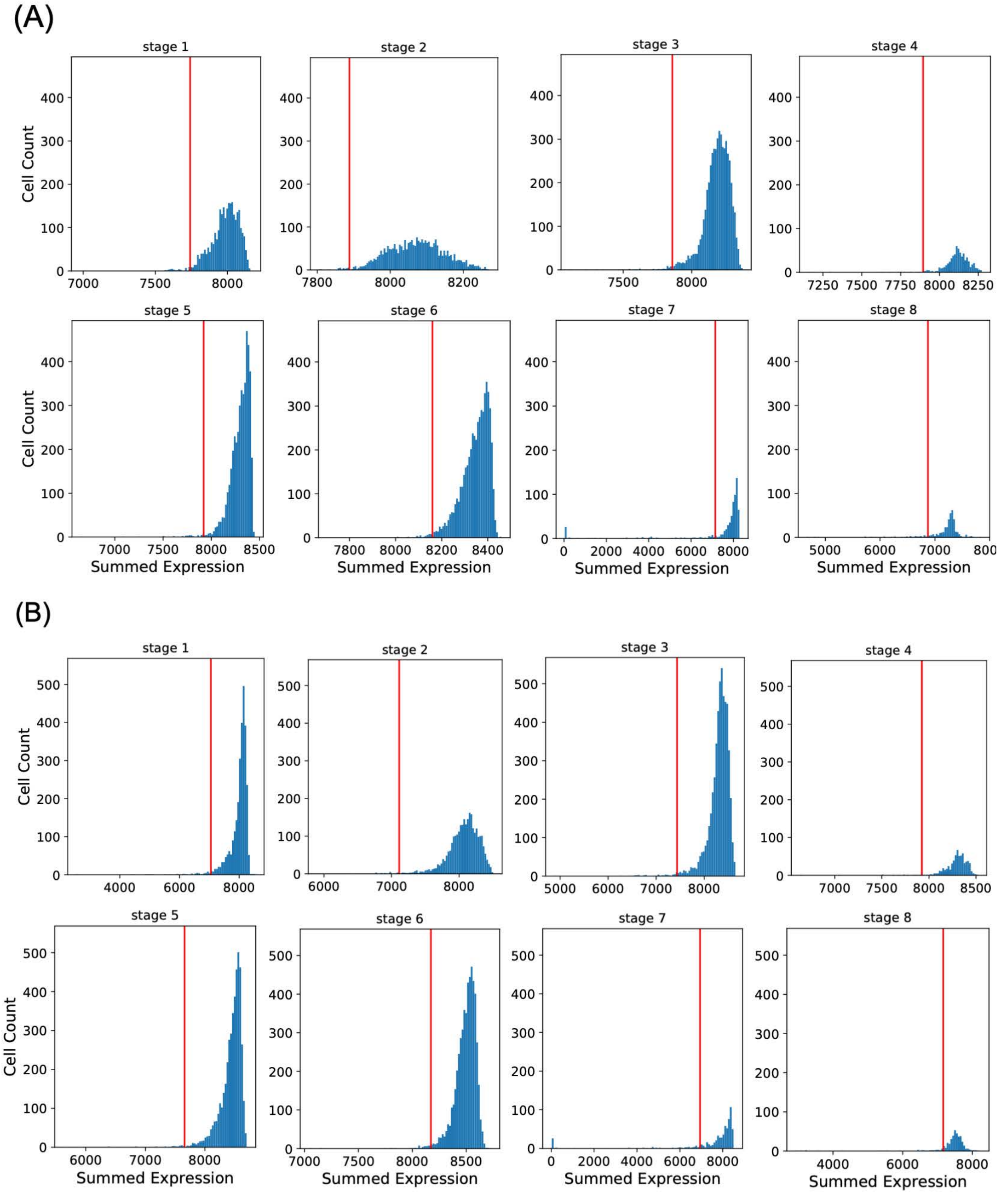

Figure S9: (A) Histogram of sum of cell imputed expression values (imputation parameter t=10). Summation is over genes expressed (greater than or equal to 1 raw UMI) in at least 1 percent of all keratinocytes and is intended to identify outlier cells with unusually low expression of all typical genes. Red vertical lines indicate thresholds on summed expression; cells with summed expression below threshold were excluded from all analysis downstream of cell clustering. (B) same as for (A) but with imputed expression values calculated using imputation parameter t=4.

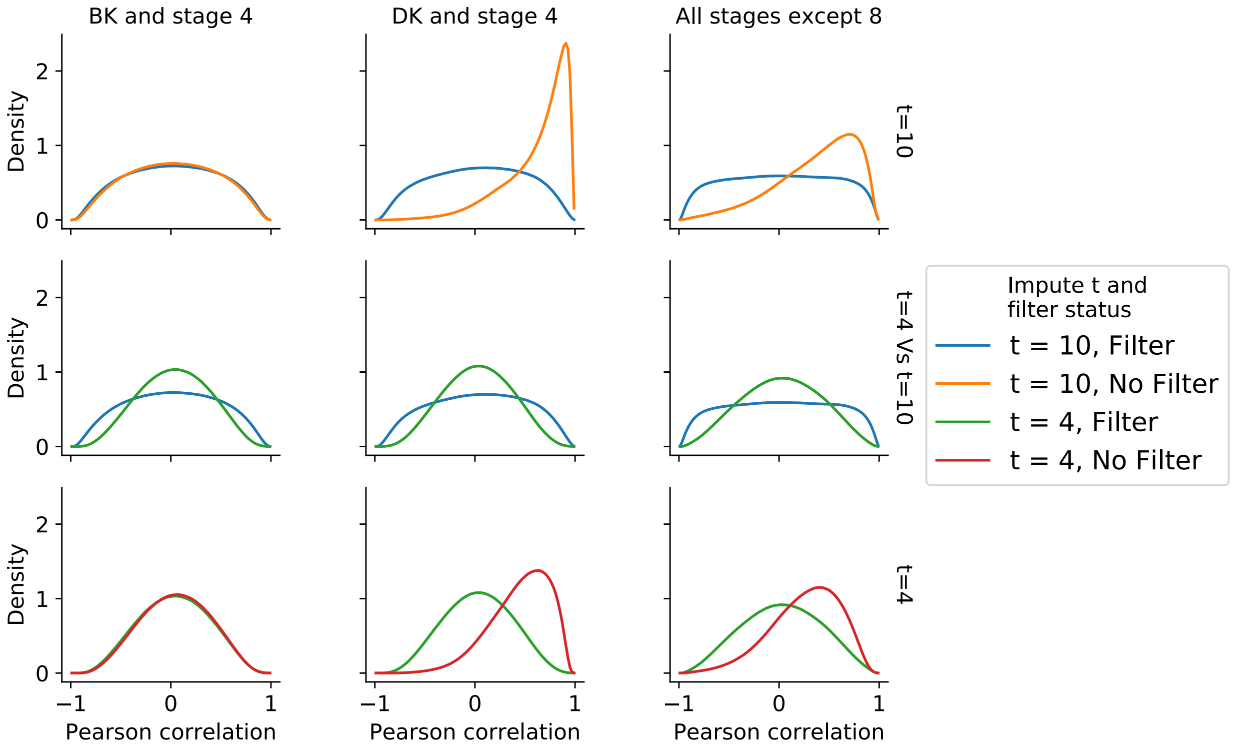

Figure S10: Effect of outlier filtering and imputation t parameter on distribution of pairwise gene correlations. Columns distinguish sets of cells used for calculating correlations: cells in stages stages 1-4 (first column), cells in stages stages 4-7 (second column), cells in stages 1-7 (third column) . Rows compare methods for calculating correlations. The first row shows that filtering of outlier cells prevents skew in distribution of correlations. Second row shows that reducing t parameter from 10 to 4 narrows the distribution of correlations, potentially reducing false positive regulatory relationships. Third row shows that filtering of outlier cells also prevents skew in distribution of correlations calculated using t=4.
