# Supplementary material for: Single-Cell Transcriptomics Reveals Spatial and Temporal Turnover of Keratinocyte Differentiation Regulators
Source: Front Genet. 2019 Sep 3;10:775. doi: 10.3389/fgene.2019.00775 (PMC6733986; doi:10.3389/fgene.2019.00775)
Supplement: Supplementary file 2 [file DataSheet_2.zip › Data_Sheet_2_Finnegan_et_al_Frontiers/S6_table_summedExprFilter.pdf]

	Threshold percentile	Number of cells filtered	Number of cells remaining	Threshold summed expression t=10	Threshold summed expression t=4
stage 1	2	57	2792	7740.726	7048.418
stage 2	1	29	2810	7888.127	7113.830
stage 3	1	53	5165	7857.910	7435.458
stage 4	2	14	645	7893.911	7924.719
stage 5	1	45	4378	7920.694	7658.153
stage 6	1	53	5222	8159.447	8172.413
stage 7	15	101	569	7144.000	6937.467
stage 8	10	41	364	6871.834	7162.116
