# Supplementary material for: Single-Cell Transcriptomics Reveals Spatial and Temporal Turnover of Keratinocyte Differentiation Regulators
Source: Front Genet. 2019 Sep 3;10:775. doi: 10.3389/fgene.2019.00775 (PMC6733986; doi:10.3389/fgene.2019.00775)
Supplement: Supplementary file 3 [file DataSheet_3.pdf]

Supplementary Methods and Supplementary References

SUPPLEMENTARY METHODS

Identification of keratinocyte stages

To identify clusters of functionally distinct keratinocytes, we used our previously published imputation, dimensionality reduction and spectral clustering techniques (Cheng et al., 2018). Imputation mitigates the effect of scRNA-seq dropout by sharing expression information among similar cells. We used the MAGIC imputation algorithm (version 0.0) (van Dijk et al., 2018) with cell similarity matrix obtained from ZINB-WaVE dimensionality reduction (Risso et al., 2018). In this method, ZINB-WaVE fits a model predicting the mean expression and probability of dropout for each gene in each cell from cell-level covariates (percent mitochondrial UMI, total UMI and batch) and from 20 latent cell-level covariates that are learned from the data, yielding a low dimensional, bias-corrected representation of each cell. The resulting 92889×20 matrix of low dimensional cell representations were used to calculate cell-cell distances needed for constructing the affinity matrix for MAGIC's diffusion-based imputation. Our application of MAGIC used default adaptive distance parameters *ka=10, k=30* and diffusion time *t=10* as chosen previously based on recovery of simulated dropout events (Cheng et al., 2018) (Supplementary Methods: Calculation of gene correlations). Finally, because the imputed expression values output by MAGIC are not normalized to a common cell library size, we renormalized MAGIC output for each cell to units of imputed UMI per 10,000. These imputed and renormalized expression values were used in downstream cell clustering.

Analysis downstream of imputation focused on 22,338 foreskin keratinocytes, identified based on anatomic location of samples and membership in expression-based clusters identified as keratocytes in Cheng *et al.* (Cheng et al., 2018). To identify differentiation stages within these cells, we performed principal component analysis (PCA) representing each cell by the $\log_2$-transformed (with pseudocount 1) imputed expression of a set of genes robustly expressed in the full data set (at least 5 UMI in at least 100 of the 92,889 cells passing quality control). The first 20 PCs sufficed to capture nearly all the variation in our imputed data (Figure S2), and we clustered the foreskin keratinocytes in this 20 dimensional space using an adaptive distance implementation (Cheng et al., 2018) of the k-means-based approximate spectral clustering (KASP) algorithm (Yan et al., 2009). KASP clustering with adaptive parameters *ka=10, k=30* was used to identify 8 keratinocyte clusters which were then ordered and named stages 1-8 based on mean cluster expression of known marker genes (Figure S3).

Calculation of gene correlations

Our construction of regulatory networks used co-expression, measured by Pearson correlation, as a proxy for gene-TF regulatory relationships. Pearson correlations were calculated between log-transformed imputed counts per million (cpm) using

$$\log \text{imputed cpm} = \log_{10}(100x + 1)$$

Where x denotes the output of our imputation algorithm (units of imputed counts per 10,000). We took two steps to prevent introduction of large-magnitude, spurious correlations that could lead to false positive regulatory relationships. First, we performed stage-wise filtering of cells

with outlier expression. Second, we reduced MAGIC's diffusion time parameter *t* to prevent over-smoothing of imputed expression values used to calculate correlations.

Estimates of Pearson correlation are strongly affected by outliers. In our study, these outliers were removed by filtering out cells lowly expressing genes expressed by the bulk of keratinocytes. Specifically, for each foreskin keratinocyte, we calculated the sum of imputed expression across genes expressed ($\geq$ 1 UMI raw data) in at least 1% of all keratinocytes. Stage-wise distributions of these summed expression values identified outlier cells in each stage (Figure S9); by removing cells in the lowest percentiles (see Table S6 for stage-wise thresholds), we mitigated a skew in the distribution of gene correlations (Figure S10, top row).

MAGIC's diffusion-based imputation algorithm mitigates dropout effects by replacing raw expression values with a weighted average of expression values of cells with similar low dimensional representation. The extent of local averaging increases with diffusion time *t*, and large *t* can over-smooth expression values, thereby averaging out true biological variation and thus strengthening spurious correlations. We observed this effect in the broadening of distributions of Pearson correlations calculated using $t=10$, compared to the same distribution calculated using $t=4$ (Figure S10, middle row). The diffusion time parameter $t=10$ was previously selected for this dataset based on recovery of simulated dropout events (Cheng et al., 2018), a useful metric for assuring that key expression values are not lost at the single cell level. Recognizing that the optimal value of the *t* parameter may depend on the type of downstream analysis and wishing to reduce spurious correlations, we used the expression values obtained from our imputation pipeline with $t=4$ and filtered for outliers using the above summed expression criteria (Figure S10, bottom row and Table S6) as imputed expression values in all analyses downstream of keratinocyte stage identification.

Clustering transcription factor expression trajectories and super-enhancer differential motif enrichment.

We performed hierarchical clustering of stage-wise mean expression values to identify dynamic TFs showing similar differentiation trajectories. Keratinocyte TFs (Methods: Identification of keratinocyte-specific genes and transcription factors) were filtered to include only those whose maximum value of mean imputed expression across stages 1-7 was at least 1.75-fold higher than the minimum across the same set; to discard lowly expressed TFs, the minimum was set to 5 counts per million (cpm) when it was less than this threshold. The stage-wise mean expression values of these dynamic TFs were converted to log cpm with pseudocount 1 and then clustered using Pearson correlation distance and average linkage.

To relate regulatory activity measured by TF expression to regulatory activity measured by abundance of functional TF binding sites, we performed differential motif enrichment analysis in super-enhancers (SEs) characterizing BK vs. DK states. We obtained hg19 coordinates of BK and DK SEs from the authors of Klein et al. (2017) (referred to as NHEK-P SE and NHEK-D SE in that publication) and used Bedtools (Quinlan and Hall, 2010) to define BK-specific SEs not overlapping any DK SEs and DK-specific SEs not overlapping BK SEs. Next, we collected position-specific scoring matrices associated with our Keratinocyte TFs from the JASPAR (Mathelier et al., 2016), TRANSFAC (Matys et al., 2006), and Hocomoco (Kulakovskiy et al., 2018) databases, as well as those published in Jolma et al. (2013). FIMO (version 5.0.1) (Grant et al., 2011) was used to scan BK- and DK-specific SEs with each motif using default parameters plus the max-strand option and a 0th order Markov background model given by the background frequencies of single nucleotides in the union of BK- and DK-specific

SEs. This produced a table of motif hit counts for each TF motif in each BK- or DK-specific SE. Motifs were tested for differential enrichment of hit counts per unit length between BK-specific and DK-specific SEs using the Mann-Whitney U test followed by Benjamini-Hochberg multiple hypothesis correction. We accepted motifs with adjusted p-values less than 10^{-3} as differentially enriched and used the asymptotic normality of the U statistic under the null hypothesis to measure the magnitude and direction of enrichment as the z-score of the U statistic:

$$z = \frac{U - \mu}{\sigma},$$

where μ and σ are the mean and standard deviation of U under the null (Mann and Whitney, 1947). Specifically, letting n_1 and n_2 denote the number of BK- and DK-specific SEs,

$$\mu = \frac{n_1 n_2}{2},$$

and

$$\sigma = \sqrt{\frac{n_1 n_2 (n_1 + n_2 + 1)}{12}}.$$

When motifs from multiple databases yielded differential enrichment for the same TF or TF dimer, we selected the strongest motif, by calling the length of the shortest candidate motif ℓ and then ranking the motifs by the sum of Kullback-Leibler divergence from the 0th-order background across ℓ most divergent bases. Finally, some TFs, such as JUN, FOS and FOSL1, were associated with several different motifs either as monomers or components of heterodimers; in the case of differential enrichment for these functionally distinct motifs, we assigned to the TF the mean of the U statistic z-scores for these enriched motifs.

Prioritization of knockdown targets

We prioritized Candidate Keratinocyte TFs according to log-fold change, during differentiation, of putative targets selected from the set of Keratinocyte Genes (Methods: Identification of keratinocyte specific genes and transcription factors). To identify regulatory targets, we first partitioned Candidate Keratinocyte TFs, denoted here by the set T, according to their pattern of differential expression between the BK and DK states (Methods: Differential expression). The set $T^{(BK)}$ contained TFs differentially upregulated in the BK state; the set $T^{(DK)}$ contained TFs differentially upregulated in the DK state; and, the set $T^{(nDE)}$ contained TFs not differentially expressed between the two states.

Next, we considered as potential targets the set *G* of Keratinocyte Genes differentially expressed between the BK and DK state. Activating and inhibiting relationships between elements of *T* and *G* were assigned based on the strength of correlation calculated across cells specific to each partition of T. More precisely, for TFs in the partitions $T^{(BK)}, T^{(DK)}$ and $T^{(nDE)}$, we computed correlations across cells in stages 1-4, 4-7 and 1-7, respectively, leading to the Pearson correlation coefficients $r_{i,j}^{(BK)}$, $r_{i,j}^{(DK)}$, $r_{i,j}^{(nDE)}$ between the log-transformed imputed expression of TF i and gene j (Supplementary Methods: Calculation of gene correlations). Next,

for each partition $k \in \{BK, DK, nDE\}$, we constructed thresholds, $r_+^{(k)}$ and $r_-^{(k)}$, on correlation strength using

$$r_+^{(k)} = \max(0\,, \text{percentile}(95\,, \{r_{i,j}^{(k)}: i \in T \text{ and } j \in (G \cup T) - \{i\}\}\ )\ )$$

$$r_-^{(k)} = \min(0\,, \text{percentile}(5\,, \{r_{i,j}^{(k)}: i \in T \text{ and } j \in (G \cup T) - \{i\}\}\ )\ )$$

where $\text{percentile}(x, A)$ denotes the xth percentile of the set A. Finally, each TF i in each partition $T^{(k)}$ was assigned a differentiation-promoting score, $\text{score}(i)$, by summing $\log_2$ expression fold-changes between DK and BK states for all elements of G passing the thresholds $r_+^{(k)}$ and $r_-^{(k)}$:

$$\text{score}(i) = \sum_{j \in G - \{i\}} \text{sign}\left(r_{i,j}^{(k)}\right) L(j) \left(I\left(\, r_{i,j}^{(k)} \geq r_+^{(k)}\right) + I\left(\, r_{i,j}^{(k)} \leq r_-^{(k)}\right)\right).$$

In this equation, I denotes the indicator function, $L(j)$ is the $\log_2$ fold-change of expression for gene j between the DK and BK states (Methods: Differential Expression) and $\text{sign}\left(r_{i,j}^{(k)}\right)$ accounts for the activating or inhibiting effect of TF i on gene j. Figure S4 shows the resulting differentiation-promoting scores along with $\log_2$-fold expression changes between imputed single-cell data averaged over the DK and BK states and between keratinocytes cultured in high (1.2 mM Ca) and low (0.07 mM Ca) calcium conditions. RNAi knockdown experiments tested the basal promoting function of four TFs with top five negative differentiation-promoting scores, after removing HOXA1 which was lowly expressed (less than 5 FPKM) in the keratinocytes cultured in *in-vitro* basal/proliferative conditions.

Regulatory network construction

Regulatory networks were constructed for the BK and DK states as follows. For the BK state, we considered Keratinocyte TFs with motifs enriched in BK SEs compared to DK SEs as putative BK regulators. Similarly, we took Keratinocyte Genes not downregulated in the BK state compared to the DK state as putative BK targets. Signed similarity scores $S_{i,j}$ between genes i and j were calculated using the soft thresholding method of Zhang and Horvath (2005):

$$S_{i,j} = \text{sign}\left(r_{i,j}^{(BK)}\right)\left|r_{i,j}^{(BK)}\right|^{\beta}$$

where $r_{i,j}^{(BK)}$ denotes the Pearson correlation of log-transformed imputed expression for genes i and j across single cells in stages 1-4, and $\beta = 4$. Putative BK regulators were organized by hierarchical agglomerative clustering using the distance

$$d(i,j) = 1 - S_{i,j}$$

and average linkage. TF modules were called using the "inconsistent" criteria in SciPy's fcluster function with parameters depth=2 and threshold=0.75 (Jones et al., 2001-). Putative BK target genes were also organized by hierarchical agglomerative clustering. Each target gene was represented by a vector of similarity scores between the gene and all putative BK regulators. These vectors were clustered using Euclidean distance and average linkage. Like TF modules, gene modules were called using the "inconsistent" criteria of the fcluster function with parameters depth=4 and threshold=2.15 (Figure S5A). We identified regulatory relationships

between pairs of identified Gene and TF Modules by applying thresholding to the distribution of magnitude of mean similarity scores between all pairs:

$$\left\{ \left| \underset{i \in A, j \in B}{\text{mean}} S_{i,j} \right| : \ A \in \text{TF Modules}, \text{B} \in \text{Gene Modules} \right\},$$

(Figure S5B). TF-Gene Module pairs with mean signed similarly score magnitude exceeding the threshold of Figure S5C were identified as having activating or inhibiting regulatory relationships (Figure S5D) and were the focus of further investigation.

The DK state network was constructed in the same manner as the BK state subject to the following changes: putative DK regulators were selected for motif enrichment in DK SEs compared to BK SEs; putative DK targets were Keratinocyte Genes not downregulated in the DK state compared to the BK state; calculation of Pearson correlations used single cells in stages 4-7; identification of TF modules used the fcluster function with parameters depth=2 and threshold=0.75; and identification of target gene modules used the fcluster function with parameters depth=16 and threshold=3.2 (Figure S8A-D).

Antioxidant analysis

Genes annotated for antioxidant function were downloaded from the AmiGO2 database (version 2.5.12) (Carbon et al., 2009) and filtered to include only those genes expressed in more than 1% of all keratinocytes in scRNAseq data (Table S2). Genes with dynamic expression in foreskin keratinocytes ($\log_2$ fold-change between minimum and maximum stage-wise mean expression for stages 1-7 greater than 1, with the minimum set to 5 imputed cpm when it was less than this threshold) were selected for hierarchical agglomerative clustering. We clustered genes represented as vectors of $\log_2$ stage-wise mean imputed cpm with pseudocount 1 using Pearson correlation distance and average linkage.

To test the significance of size enrichment of the cluster showing peak expression in stages 1-3, we generated a null distribution of maximum cluster sizes using a permutation approach. For each of 10,000 iterations, we independently permuted the elements of each $\log_2$ stage-wise mean expression vector and repeated the hierarchical clustering procedure identifying four clusters. The *p*-value was calculated from the percentile of the observed cluster size in the distribution of simulated maximum cluster sizes.

SUPPLEMENTARY REFERENCES

Carbon, S., Ireland, A., Mungall, C.J., Shu, S., Marshall, B., Lewis, S., et al. (2009). AmiGO: online access to ontology and annotation data. *Bioinformatics* 25(2)**,** 288-289. doi: 10.1093/bioinformatics/btn615.

Cheng, J.B., Sedgewick, A.J., Finnegan, A.I., Harirchian, P., Lee, J., Kwon, S., et al. (2018). Transcriptional Programming of Normal and Inflamed Human Epidermis at Single-Cell Resolution. *Cell Rep* 25(4)**,** 871-883. doi: 10.1016/j.celrep.2018.09.006.

Grant, C.E., Bailey, T.L., and Noble, W.S. (2011). FIMO: scanning for occurrences of a given motif. *Bioinformatics* 27(7)**,** 1017-1018. doi: 10.1093/bioinformatics/btr064.

Jolma, A., Yan, J., Whitington, T., Toivonen, J., Nitta, K.R., Rastas, P., et al. (2013). DNA-binding specificities of human transcription factors. *Cell* 152(1-2)**,** 327-339. doi: 10.1016/j.cell.2012.12.009.

Jones, E., Oliphant, T., Peterson, P., and others (2001-). "SciPy: Open source scientific tools for Python". 1.0.0 ed.).

Klein, R.H., Lin, Z., Hopkin, A.S., Gordon, W., Tsoi, L.C., Liang, Y., et al. (2017). GRHL3 binding and enhancers rearrange as epidermal keratinocytes transition between functional states. *PLoS Genet* 13(4)**,** e1006745. doi: 10.1371/journal.pgen.1006745.

Kulakovskiy, I.V., Vorontsov, I.E., Yevshin, I.S., Sharipov, R.N., Fedorova, A.D., Rumynskiy, E.I., et al. (2018). HOCOMOCO: towards a complete collection of transcription factor binding models for human and mouse via large-scale ChIP-Seq analysis. *Nucleic Acids Res* 46(D1)**,** D252-D259. doi: 10.1093/nar/gkx1106.

Mann, H.B., and Whitney, D.R. (1947). On a test of whether one of two random variables is stochastically larger than the other. *The Annals of Mathematical Statistics* 18(1)**,** 50–60

Mathelier, A., Fornes, O., Arenillas, D.J., Chen, C.Y., Denay, G., Lee, J., et al. (2016). JASPAR 2016: a major expansion and update of the open-access database of transcription factor binding profiles. *Nucleic Acids Res* 44(D1)**,** D110-115. doi: 10.1093/nar/gkv1176.

Matys, V., Kel-Margoulis, O.V., Fricke, E., Liebich, I., Land, S., Barre-Dirrie, A., et al. (2006). TRANSFAC and its module TRANSCompel: transcriptional gene regulation in eukaryotes. *Nucleic Acids Res* 34(Database issue)**,** D108-110. doi: 10.1093/nar/gkj143.

Quinlan, A.R., and Hall, I.M. (2010). BEDTools: a flexible suite of utilities for comparing genomic features. *Bioinformatics* 26(6)**,** 841-842. doi: 10.1093/bioinformatics/btq033.

Risso, D., Perraudeau, F., Gribkova, S., Dudoit, S., and Vert, J.P. (2018). A general and flexible method for signal extraction from single-cell RNA-seq data. *Nat Commun* 9(1)**,** 284. doi: 10.1038/s41467-017-02554-5.

van Dijk, D., Sharma, R., Nainys, J., Yim, K., Kathail, P., Carr, A.J., et al. (2018). Recovering Gene Interactions from Single-Cell Data Using Data Diffusion. *Cell* 174(3)**,** 716-729 e727. doi: 10.1016/j.cell.2018.05.061.

Yan, D.H., Huang, L., and Jordan, M.I. (2009). Fast Approximate Spectral Clustering. *Kdd-09: 15th Acm Sigkdd Conference on Knowledge Discovery and Data Mining***,** 907-915.

Zhang, B., and Horvath, S. (2005). A general framework for weighted gene co-expression network analysis. *Stat Appl Genet Mol Biol* 4**,** Article17. doi: 10.2202/1544-6115.1128.
